# Long-term stability of computational parameters during approach-avoidance conflict in a transdiagnostic psychiatric patient sample

**DOI:** 10.1038/s41598-021-91308-x

**Published:** 2021-06-03

**Authors:** Ryan Smith, Namik Kirlic, Jennifer L. Stewart, James Touthang, Rayus Kuplicki, Timothy J. McDermott, Samuel Taylor, Sahib S. Khalsa, Martin P. Paulus, Robin L. Aupperle

**Affiliations:** Laureate Institute for Brain Research, 6655 S Yale Ave, Tulsa, OK 74136 USA

**Keywords:** Computational neuroscience, Human behaviour, Anxiety, Depression, Addiction

## Abstract

Maladaptive behavior during approach-avoidance conflict (AAC) is common to multiple psychiatric disorders. Using computational modeling, we previously reported that individuals with depression, anxiety, and substance use disorders (DEP/ANX; SUDs) exhibited differences in decision uncertainty and sensitivity to negative outcomes versus reward (emotional conflict) relative to healthy controls (HCs). However, it remains unknown whether these computational parameters and group differences are stable over time. We analyzed 1-year follow-up data from a subset of the same participants (*N* = 325) to assess parameter stability and relationships to other clinical and task measures. We assessed group differences in the entire sample as well as a subset matched for age and IQ across HCs (*N* = 48), SUDs (*N* = 29), and DEP/ANX (*N* = 121). We also assessed 2–3 week reliability in a separate sample of 30 HCs. Emotional conflict and decision uncertainty parameters showed moderate 1-year intra-class correlations (.52 and .46, respectively) and moderate to excellent correlations over the shorter period (.84 and .54, respectively). Similar to previous baseline findings, parameters correlated with multiple response time measures (*p*s < .001) and self-reported anxiety (*r* = .30, *p* < .001) and decision difficulty (*r* = .44, *p* < .001). Linear mixed effects analyses revealed that patients remained higher in decision uncertainty (SUDs, *p* = .009) and lower in emotional conflict (SUDs, *p* = .004, DEP/ANX, *p* = .02) relative to HCs. This computational modelling approach may therefore offer relatively stable markers of transdiagnostic psychopathology.

## Introduction

Situations eliciting conflict between approach and avoidance drives (approach-avoidance conflict; AAC) are often difficult to handle adaptively for individuals with affective psychopathology^1^. For example, maladaptive avoidance behavior in depression and anxiety disorders (DEP/ANX) can lead individuals to sacrifice participation in rewarding activities due to fear of negative social consequences^2^. Substance use disorders (SUDs) are also characterized by conflict between strong drug-taking drives and long-term negative outcomes (job loss, loss of close relationships), and decision-making impairments are often observed in substance users on tasks that involve AAC (reviewed in^3,4^). The ability to identify stable individual differences in cognition during AAC in these and other clinical populations could be an important step toward the development of individualized treatment selection^5^.

While there is a growing literature using multiple behavioral tasks to study AAC (^6–18^; for a review, see^19^), this work has not focused on longitudinal aspects of AAC behavior in clinical populations. It is also unclear whether repeated testing of participants with existing behavioral tasks would provide stable individual difference estimates in psychiatric patients. Yet, individual test–retest reliability and stability of group differences are essential components for any paradigm to be used in assessing the effects of treatment (e.g., to assess whether a clinical intervention reduces an objective measure of avoidance behavior). Behavioral paradigms capable of offering stable, clinically relevant measures would therefore be an important advance.

In a recent paper^20^, we used a computational modelling approach to analyze behavior on an AAC task developed by Aupperle and colleagues^11^ in a transdiagnostic sample of patients with DEP/ANX and SUDs. This dataset was part of the Tulsa 1000 (T1000) study^21^, a naturalistic longitudinal study recruiting subjects based on the dimensional NIMH Research Domain Criteria framework^22^. Computational modelling in that study was able to tease apart two components of AAC: decision uncertainty and emotional conflict (aversiveness of negative stimuli relative to reward value). Relative to healthy controls (HCs), we found that all patient groups showed elevated decision uncertainty and also exhibited an unexpected trend toward reduced levels of emotional conflict. Behaviorally, this corresponded to greater choice variability, but less pronounced avoidance on average, in the patient groups. However, the stability of this difference in the patient groups over time, and the ability of the task and modelling approach to capture it, was not addressed.

A central aim in computational psychiatry is to find modeling measures that can be used as predictors of treatment outcome, to inform personalized medicine approaches, or to track progress in treatment. These aims require that such measures provide reliable estimates of functioning in the domain being assessed. In contrast, if changes with repeated testing reflect random influences, and not change in an underlying mechanism of interest, their use as assessment tools for tracking progress in treatment will be limited. To date there have been relatively few assessments of the test–retest reliability of computational psychiatry measures, with existing studies finding reliability levels ranging from poor to good^23–29^. This pattern of results suggests that either the cognitive processes engaged by these tasks differ with repeated performance or that there is significant measurement error. There is thus a need to more fully assess the longitudinal reliability of available task measures within computational psychiatry research.

In this paper, we perform identical analyses on data from participants in our previous report ^20^who performed the AAC task a second time at a 1-year follow-up. We tested the hypothesis that model parameter estimates would show at least fair reliability over the 1-year period [i.e., intra-class correlations greater than 0.40; criteria for ‘fair’ vs. ‘good’ (greater than 0.60) reliability are based on^30^] and that the same differences between healthy and clinical groups would be found as in the baseline visit. We also explored whether baseline parameter estimates could predict changes in symptoms between baseline and 1-year follow-up. Lastly, we also report test–retest reliability of parameters over a 2–3 week period for a separate sample of 30 healthy control participants.

## Method

### Participants

Participants for this analysis were identified from the first 500 participants of the Tulsa 1000 (T1000)^21^, a naturalistic longitudinal study that recruited participants based on the dimensional NIMH Research Domain Criteria framework. The T1000 study had a baseline visit and a 1-year follow up, and included a community-based sample of approximately 1000 individuals recruited through radio, electronic media, treatment center referrals, and word of mouth (this sample size was planned a priori; see^21^ for a detailed justification based on the aims of the larger study). The participants included in the analyses described here consisted of the subset of participants in our previous study^20^ that returned for the 1-year follow-up visit (see below). These participants were 18–55 years of age, and were screened on the basis of dimensional psychopathology scores: Patient Health Questionnaire (PHQ^31^) ≥ 10, Overall Anxiety Severity and Impairment Scale (OASIS^32^) ≥ 8, and/or Drug Abuse Screening Test (DAST-10^33^) score ≥ 3. HCs did not show elevated symptoms or psychiatric diagnoses. Participants were excluded if they (i) tested positive for drugs of abuse, (ii) met criteria for psychotic, bipolar, or obsessive–compulsive disorders, or reported (iii) history of moderate-to-severe traumatic brain injury, neurological disorders, or severe or unstable medical conditions, (iv) active suicidal intent or plan, or (v) change in medication dose within 6 weeks. Full inclusion/exclusion criteria are described in^21^. The study was approved by the Western Institutional Review Board. All participants provided written informed consent prior to completion of the study protocol, in accordance with the Declaration of Helsinki, and were compensated for participation. ClinicalTrials.gov identifier: #NCT02450240. For previous papers published from the larger T1000 dataset, see^34–44^. With the exception of our previous paper^20^, none of these papers included analyses using the AAC task.

As in our original study^20^, given the heterogeneous clinical sample in the T1000 and its explicitly transdiagnostic focus, we divided participants into three groups consisting of HCs, those with SUDs (with low to moderate DEP/ANX symptoms; see Table 1), and those with DEP/ANX who did not have SUDs. This maintained the transdiagnostic focus while also allowing us to account for potentially distinct effects of substance use. Diagnostic grouping was based on DSM-IV or DSM-5 criteria using the Mini International Neuropsychiatric Inventory (MINI)^45^ administered at the baseline visit. The DEP/ANX group consisted of individuals with major depression and/or co-morbid anxiety disorders (social anxiety, generalized anxiety, panic, and/or posttraumatic stress disorder; baseline: *N* = 260, 1-year follow-up: *N* = 192), SUDs (recreational drugs excluding alcohol and nicotine, with or without comorbid depression and anxiety disorders; baseline: *N* = 159, 1-year follow-up: *N* = 84), and HCs with no mental health diagnoses (baseline: *N* = 59, 1-year follow-up: *N* = 49). In total, 68% of participants completed the follow-up visit (for analyses of potential symptom differences between those that did versus did not complete the follow-up visit, see Supplementary Materials).

**Table 1:** Summary statistics (mean and SD) and group differences for demographic and clinical measures.

Full sample	HCs (N = 49)	DEP/ANX (N = 192)	SUDs (N = 84)	p*
Age	32.71 (11.29)	37.17 (11.42)	36.28 (9.18)	0.039
Sex (% male)	24 (49.0%)	50 (26.0%)	34 (40.5%)	0.002
PHQ baseline	0.85 (1.27)	12.57 (5.19)	6.88 (6.10)	< 0.001
PHQ 1-year follow-up	1.14 (1.84)	8.27 (6.10)	3.10 (4.57)	< 0.001
OASIS baseline	1.38 (2.00)	9.74 (3.47)	5.98 (4.86)	< 0.001
OASIS 1-year follow-up	1.47 (2.31)	7.56 (4.58)	3.54 (4.36)	< 0.001
DAST-10 Baseline	0.12 (0.39)	0.56 (1.27)	7.46 (2.21)	< 0.001
DAST-10 1-year follow-up	0.47 (0.56)	0.56 (1.05)	2.46 (2.89)	< 0.001
WRAT baseline	63.89 (4.54)	63.03 (4.64)	58.49 (6.00)	< 0.001

Matched sample	HCs (N = 48)	DEP/ANX (N = 121)	SUDs (N = 29)	*p*
Age	32.94 (11.29)	36.46 (11.13)	34.06 (9.93)	0.144
Sex (% male)	24 (50.0%)	31 (25.6%)	13 (44.8%)	0.005
PHQ baseline	0.85 (1.27)	12.57 (5.34)	8.69 (7.49)	< 0.001
PHQ 1-year follow-up	1.14 (1.84)	8.10 (6.44)	4.03 (4.12)	< 0.001
OASIS baseline	1.38 (2.00)	9.71 (3.44)	6.93 (5.52)	< 0.001
OASIS 1-year follow-up	1.47 (2.31)	7.26 (4.53)	4.52 (4.40)	< 0.001
DAST-10 Baseline	0.12 (0.39)	0.53 (1.18)	7.52 (2.69)	< 0.001
DAST-10 1-year follow-up	0.47 (0.56)	0.56 (1.18)	2.69 (2.97)	< 0.001
WRAT baseline	63.89 (4.54)	62.88 (4.60)	61.59 (5.34)	0.123

*Based on ANOVAs testing for the presence of differences between groups.

As further described in^21^, because the T1000 study was built around the NIMH Research Domain Criteria (RDoC) framework that describes dimensions of pathology^22^, it specifically aimed in advance to recruit participants with these symptom profiles, with the aim of identifying transdiagnostic behavioral and neural phenotypes related to threat/reward processing, interoceptive processing, and cognitive functioning. While symptoms can be observed dimensionally, as in the case of symptom scales, we found that diagnostic categories offered additional information in our previous study; they also allow more direct connection to previous diagnosis-based studies. The transdiagnostic categories used here (i.e., separating SUDs from DEP/ANX without SUDs) were developed prior to the current analyses and discussed in previous papers^34^. Note also that, while the T1000 also included individuals with eating disorders, these were excluded in our original study (and here) due to small sample sizes. Depression and anxiety disorders were categorized together for our analyses due to the high rates of overlap in these diagnoses and due to there being very small sample sizes (*N* = 19) for individuals with anxiety disorders without depression. The lower number of HCs was included to maximize our ability to detect dimensional effects within patient populations in other planned analyses (in consideration of the total sample size that could be collected).

### Data collection procedure

Participants underwent an intensive assessment for demographic, clinical and psychiatric features, with a main focus on negative and positive affect, arousal, and cognitive functioning. From this assessment, several direct and derived variables were acquired, only some of which were used in the present analyses. The complete list of assessments and references supporting their validity and reliability are provided in^21^. For this study, we examined the following additional measures that were collected at both baseline and follow-up: the Patient-Reported Outcomes Measurement Information System (PROMIS) depression and anxiety scales^46^, the Behavioral Activation/Inhibition (BIS/BAS) scales^47^, the PANAS positive and negative affect scales^48^, and the anxiety sensitivity index (ASI;^49^).

### Approach-avoidance conflict (AAC) task

**Figure 1:**
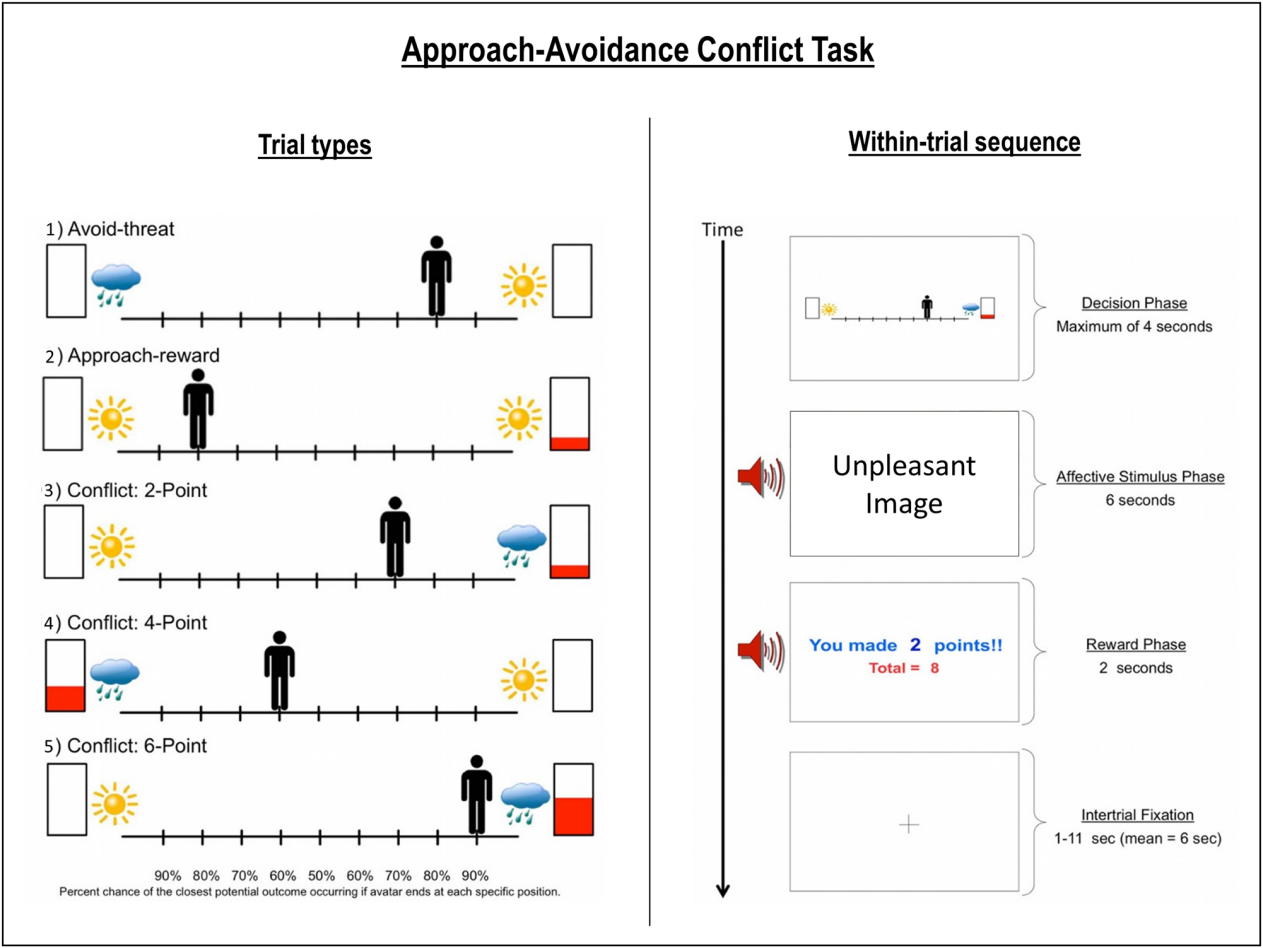
(Left) The five trial types. The sun indicates a positive stimulus, the cloud indicates a negative stimulus, and the higher the red bar is filled the more points may be received. (Right) Example trial in the AAC task, in which the negative stimulus and two points were presented based on the probabilities associated with the chosen runway position. This figure is modified from our previous paper^20^.

The AAC task was described in detail in our previous paper (and elsewhere^10,11,20^). For a more detailed description, see Supplementary Materials. Briefly, participants were shown an avatar on a runway which could be moved to 9 possible locations, with images of a sun, raincloud, and/or a rectangle with partial red fill on either side (depending on the task condition; see Fig. 1). They could choose to approach the sun or clouds, where moving closer to one or the other increased the probability of exposure to a pleasant or unpleasant image/sound combination (respectively), with probabilities of *p* = 0.1 to 0.9 in increments of 0.1 (which was indicated to participants). The higher the red fill on a given side, the more points they could win if they approached that side.

There were five trial types: (1) ‘Avoid-threat’ (AV), in which 0 points were offered for both negative and positive stimulus outcomes; (2) ‘Approach-reward’ (APP), in which 2 versus 0 points were offered, each with positive affective stimuli. (3)–(5) Three levels of ‘Conflict’ in which the negative affective stimulus was presented in addition to winning either 2 (CONF2), 4 (CONF4), or 6 (CONF6) points, while 0 points were offered for the positive stimulus. The task consisted of a total of 60 trials, with 12 of each of the five trial types. Behavioral variables consisted of both chosen avatar position and response times (RTs; i.e., time to initial button press) during each trial. Participants were also asked to fill out the same post-task Likert scale questionnaire as in our previous study, which asked about their experience during the task (these questions are listed in Table 4 within the results section).

### Computational modeling

**Figure 2:**
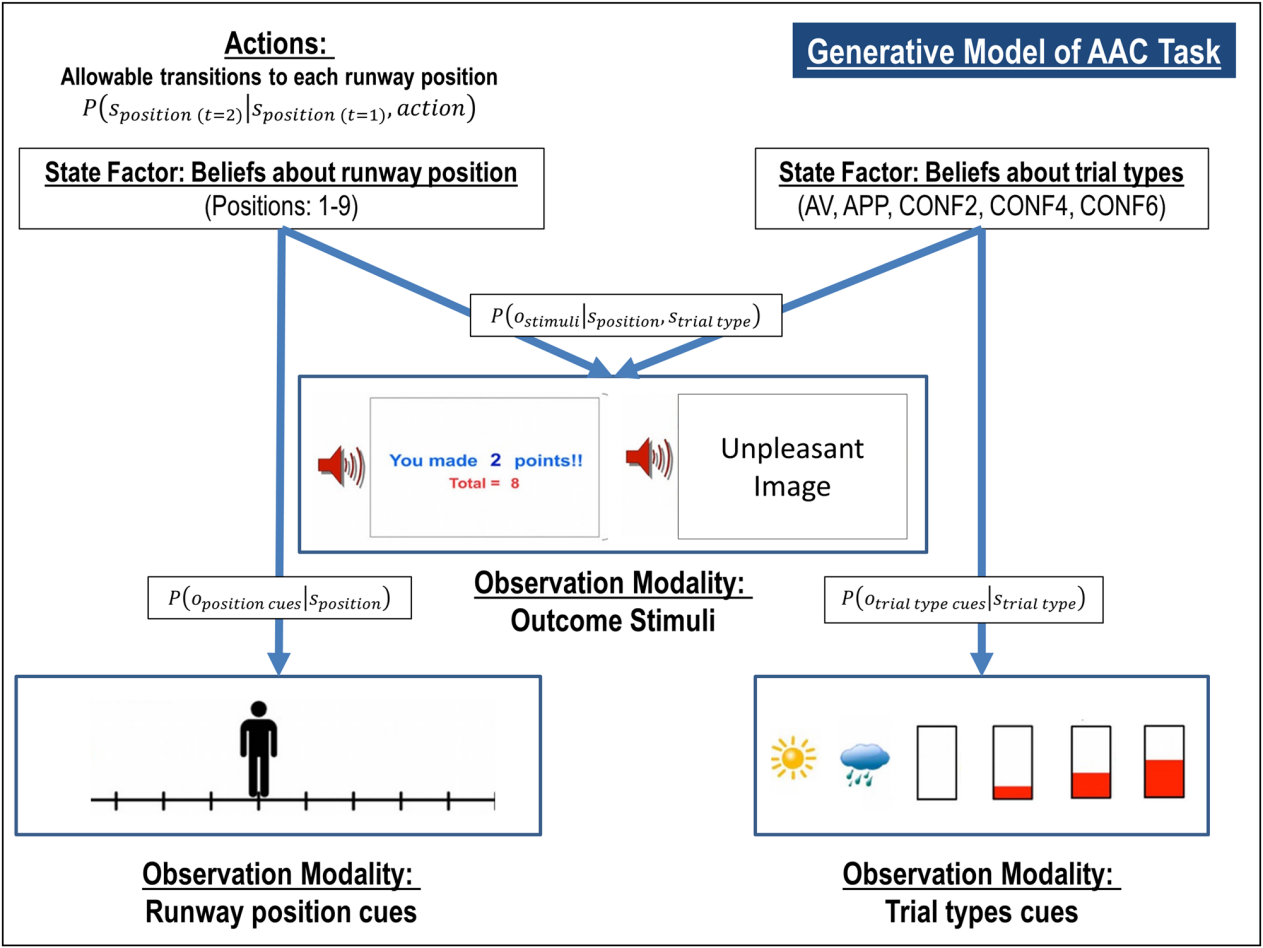
Simplified visual depiction of relevant dependencies in the computational (generative) model of the approach-avoidance conflict task. Beliefs about trial type and beliefs about runway positions were generated by (and inferred based on) trial type cues and runway position cues, respectively. Observed outcome stimuli were probabilistically generated by an interaction between trial type and runway position. Beliefs about this interaction were used to infer the action (state transition) most likely to produce the most preferred outcome stimuli. Trial Types: AV = Avoid; APP = Approach; CONF2, CONF4, and CONF6 indicate Conflict + 2 Points, 4 Points, or 6 Points, respectively. This figure is modified from our previous paper^20^.

To model behavior on the AAC task described above, we used the same Markov decision process (MDP) model as in our previous study; for more details about this class of models, see^51–54^. For a detailed description of our model specification, which was identical to our previous study, see Supplementary Materials. The model is also described in Table 2 and illustrated in Fig. 2. Briefly, the model included observable task stimuli (*o*; runway cues, trial type cues, affective stimuli, points), task states (*s*; runway position, trial type), and action policies ($\pi$; choice of runway position). Different matrices specified the probability that runway positions would lead to observing different task stimuli, $P({o}_{t}|{s}_{t})$, depending on actions, $P({s}_{t+1}|{s}_{t},\pi )$, and the subjective preference for (or aversion to) different task stimuli. An expected precision term (β) encoded decision uncertainty, and an “emotion conflict” (EC) term encoded how subjectively aversive negative stimuli were relative to point values. Higher values for the decision uncertainty term have the effect of increasing the variance of the distribution encoding the probability of selecting one action over others. This means that, all else being equal, individuals that make less consistent choices within each trial type will be assigned higher β values (e.g., choosing to approach the negative stimulus sometimes but not others when the same number of points could be won). Higher values for the EC term have the effect of increasing avoidance. This means that, all else being equal, individuals that choose to avoid the negative stimuli when more points could be won will have higher EC values (e.g., only choosing to approach the negative stimuli when 6 points could be won vs. choosing to approach when both 4 or 6 points could be won). For example simulations of behavior under different parameter values, see Figure S1 in Supplementary Materials.

**Table 2:** Markov decision process model of the AAC task.

Model variable	General definition	Model-specific specification
*o*_*t*_	Observable outcomes at time t*	Outcome modalities 1. Observed position on the runway (10 possible observations, including a “starting” position and the nine final positions on the runway that could be chosen) 2. Cues indicating trial type (five possible observations, corresponding to the five trial types) 3. Stimuli observed at the end of each trial. This included seven possible observations corresponding to a “starting” observation, the positive stimulus with 0 or 2 points, and the negative affective stimulus with 0, 2, 4, or 6 points
*s*_*t*_	Beliefs about hidden states at time *t*	Hidden state factors 1. Beliefs about position on the runway (10 possible belief states with an identity mapping to the observations in outcome modality #1) 2. Beliefs about the trial type (corresponding to the five trial types)
π	A distribution over action policies encoding the probability of choosing each policy	Allowable policies included the decision to transition from the starting state to each of the nine possible positions on the runway
*β*	The prior on expected policy precision ($\beta$) is the 'rate' parameter of a gamma distribution, which is a standard distribution to use as a prior for expected precision ($\gamma$). This latter term modulates the influence of expected free energy on policy selection	When $\beta$ is high (reflecting low confidence about the best decision), policy selection becomes less deteriministic. Higher $\beta$ values therefore encode participants’ decision uncertainty during the task (c.f., the temperature parameter in a conventional softmax response function)
**A** matrix $P{(}o_{t} {|} s_{t} )$	A matrix encoding beliefs about the relationship between hidden states and observable outcomes (i.e., the likelihood that specific outcomes will be observed given specific hidden states)	Encodes beliefs about the relationship between position on the runway and the probability of observing each outcome, conditional on beliefs about the task condition
**B** matrix $P{(}s_{t + 1} {|} s_{t} )$	A matrix encoding beliefs about how hidden states will evolve over time (transition probabilities)	Encodes beliefs about the way participants could choose to move the avatar, as well as the belief that the task condition will not change within a trial
**C** matrix $lnP\left( {o_{t} } \right)$	A matrix encoding the degree to which some observed outcomes are preferred over others (technically modeled as prior expectations over outcomes). The values for each column in this matrix are passed through a softmax function to generate a proper probability distribution, which is then log-transformed	Encodes stronger positive preferences for receiving higher amounts of points, and negative preferences for the aversive stimuli (both relative to an anchor value of 0 for the “safe” positive stimulus). The **EC** parameter in our model encodes the value of participants’ preferences against observing the aversive stimuli
**D** matrix $P\left( {s_{1} } \right)$	A matrix encoding beliefs about (a probability distribution over) initial hidden states	The simulated agent always begins in an initial starting state, and believes each task condition is stable across each trial

*Note that *t* here refers to a timepoint in each trial *about* which participants have beliefs. Before a participant makes a choice (i.e., when still in the “start” state), they have prior beliefs about the state at time *t* = 2, and these beliefs are then updated after a subsequent observed outcome. In the active inference literature these beliefs *about* timepoints are often instead denoted with the Greek letter tau (τ) in order to distinguish them from the times (*t*) *at* which new observations are presented (for details, see^54^).

All model simulations were implemented using standard routines (here spm_MDP_VB_X.m) that are available as Matlab code in the latest version (Jan 13th, 2020 update) of SPM12 academic software: http://www.fil.ion.ucl.ac.uk/spm/. Matlab code specifying the generative model of the AAC task is included as an appendix in our original paper^20^; additional code used to estimate parameters is also included as Supplementary Materials. As in our previous study, we used Variational Bayes (standard variational Laplace^55^) to estimate model parameters for each participant that maximized the likelihood of each participant’s responses, as described in^17^; see Supplementary Materials for more details. To confirm that parameters were recoverable, we also generated simulated task behavior under 46 representative parameter value combinations present in our participant behavior; we then used Variational Bayes to estimate parameter values from this simulated behavior. Pearson correlations between the true and estimated parameter values were strong (decision uncertainty: *r* = 0.94; EC: *r* = 0.9), indicating that model parameters could be accurately estimated.

### Analysis of model parameters

Test–retest reliability of individual parameter estimates at baseline and 1-year follow-up was estimated using single-measure consistency intraclass correlations [ICC(3, 1)], which account for fixed effects across time. This ICC measure was chosen due to the expectation that time and/or task familiarity could plausibly influence task behavior across all participants (although we note that the results reported below were nearly identical if assessing agreement instead of consistency). To further assess stability of results over time, we then re-performed the same analyses as in the original paper for the one year follow-up data. Specifically, we first calculated model accuracy metrics, reflecting (1) the average probability of participants’ actions under the model and (2) the average percentage of trials during which the action with the highest probability in the model matched the action chosen by participants (i.e., under the parameter values estimated for each participant). Next, we examined correlations between model parameters and RTs (i.e., time to initial button press; both across the whole task and within each condition). We also conducted correlation analyses to examine whether each parameter could predict subsequent self-reports on the post-task Likert scale questions.

We then conducted linear mixed effects analyses (LMEs) to confirm the effect of group in each parameter observed in our original study, and to assess potential effects of time (and their interaction). However, in our original study (and as shown in Table 1) the groups showed significant differences in age, sex, and Wide Range Achievement Test reading scores (WRAT; a common measure of premorbid IQ^56^), which prevented strong conclusions (i.e., even if controlling for these variables; see^57^ for discussion of limitations in the interpretability of results when controlling for effects of variables for which there are group differences). To more rigorously assess group differences, our previous study used the fullmatch function within the optmatch R package (https://www.rdocumentation.org/packages/optmatch/versions/0.9-10/topics/fullmatch; R version 4.0.2; originally developed by researchers at the Laureate Institute for Brain Research) to propensity match groups based on age and WRAT scores (propensity matching was not effective when including sex, given the differences between groups). Here, we similarly performed between-group analyses in the propensity matched groups used at baseline (i.e., the subset of participants in the original groups that returned for the follow-up visit; note that age and WRAT scores remained matched despite dropout, as shown in Table 1). Sample sizes for this matched sample (after drop-out between baseline and follow-up) were *N* = 48 (HCs), *N* = 121 (DEP/ANX), and *N* = 29 (SUDs). In Supplementary Materials, we present analogous results in the full sample, and we note below whether effects in the matched sample were also present in this larger sample. In these analyses, we expected to again observe greater β values (relative to HCs) in both patient groups at the 1-year follow up, as well as attenuated EC in SUDs. As these were a priori hypotheses focused specifically on confirming our prior results, we did not correct for multiple comparisons.

Excluding HCs, we next tested in exploratory analyses whether baseline parameter estimates could predict changes in clinical presentation between baseline and 1-year follow-up. Specifically, we used linear regressions to test whether changes in dimensional measures could be predicted (PHQ, OASIS, DAST, PROMIS depression/anxiety, BIS/BAS, PANAS positive/negative affect, ASI), after accounting for scores at baseline as well as age, sex, and WRAT scores. For purposes of hypothesis generation, in Supplementary Materials we also describe further exploratory analyses of potential predictive relationships between other task measures and a number of clinical measures available in the T1000 dataset.

### Analysis of descriptive task measures

We performed analogous analyses on descriptive task measures, including RTs and mean chosen runway positions. Test–retest reliability of these measures at baseline and 1-year follow-up was estimated using single-measure consistency intraclass correlations [ICC(3, 1)]. To assess group differences, we conducted analogous LMEs with these measures as the dependent variables.

### Follow-up analysis of short-term test–retest reliability

To further clarify test–retest reliability for the computational parameters, we estimated model parameters from a second test–retest dataset previously acquired to assess descriptive measures of the AAC task (described in^58^; also see Supplementary Materials). This dataset included 30 healthy participants (age: mean = 28.9, SD = 8.14; 19 female) who completed the task two times, approximately 2–3 weeks apart. Identical computational and ICC analyses as described above were carried out on this second sample.

### Ethical approval

All experimental protocols were approved by the Laureate Institute for Brain Research and the Western Institutional Review Board. All methods were carried out in accordance with relevant guidelines and regulations in accordance with the 1964 Helsinki declaration and its later amendments.

## Informed consent

Informed consent was obtained from all individual participants included in the study.

## Results

Descriptive statistics for demographic and clinical measures are shown in Table 1. The descriptive statistics for each of the parameters are shown in Table 3. The EC and β parameters were correlated at *r* = 0.19, *p* < 0.001. Because the parameters were not normally distributed, they were log-transformed for all subsequent analyses using the R package optLog (https://github.com/kforthman/optLog) to find the optimal log transform that minimizes skew.

**Table 3:** Summary statistics (mean and SD) and group differences for computational measures.

Full sample	HCs (N = 49)	DEP/ANX (N = 192)	SUDs (N = 84)
Decision uncertainty (β) Baseline	2.80 (2.56)	4.71 (4.95)	5.01 (4.63)
Decision uncertainty (β) 1-year follow-up	2.33 (2.48)	3.46 (3.92)	4.60 (5.29)
Emotional conflict (EC) Baseline	3.28 (2.76)	3.08 (2.85)	2.06 (2.14)
Emotional conflict (EC) 1-year follow-up	4.47 (3.66)	3.30 (3.42)	1.99 (2.16)

Matched sample	HCs (N = 48)	DEP/ANX (N = 121)	SUDs (N = 29)
Decision uncertainty (β) Baseline	2.80 (2.56)	4.73 (5.22)	4.71 (5.66)
Decision uncertainty (β) 1-year follow-up	2.36 (2.49)	3.35 (4.00)	3.74 (5.03)
Emotional conflict (EC) Baseline	3.28 (2.76)	3.23 (3.07)	2.18 (2.39)
Emotional conflict (EC) 1-year follow-up	4.43 (3.68)	3.27 (3.37)	2.35 (2.75)

### Intraclass correlations

Across all participants, the ICC between baseline and 1-year follow-up was fair for both β (ICC = 0.46; *F*(323, 323) = 2.68,* p* < 0.001) and EC values (ICC = 0.52 (*F*(323, 323) = 3.2,* p* < 0.001). Similar results were found when examining groups separately (see Supplementary Materials).

As detailed in Supplementary Materials, ICCs across participants, and by group, also showed fair to good reliability in each of the five task conditions for both RTs (ICCs between 0.5 and 0.61) and mean chosen runway position (ICCs between 0.4 and 0.56); with the exception that, in HCs, reliability of mean chosen runway position in the AV and APP conditions was poor (ICC = 0.23 and − 0.03, respectively). ICCs for dimensional clinical measures also showed fair to good reliability over the 1-year period (ICCs between 0.56 and 0.70; with the exception of the DAST, with ICC = 0.43; see Supplementary Materials).

### Face validity: task-related self-report and behavior

Averaging across participants, model accuracy at 1-year follow-up was 81% (SE = 1.3%; note: chance accuracy is 1/9 = 11%). The average probability of participants’ actions under the model was *p* = 0.68 (SE = 0.02). Confirming our prior result at baseline, individuals with slower RTs in each trial type exhibited higher β values (*r* = 0.33 for the AV condition; *r* = 0.55–0.58 for the other four conditions,* p* < 0.001 each). Higher EC values were associated with slower RTs in the three conflict conditions (*r* = 0.17–0.20,* p* < 0.001 each) and faster RTs in the AV condition (i.e., individuals with greater sensitivity to the unpleasant stimuli, as indicated by higher EC, responded faster during AV trials; *r* = − 0.28,* p* < 0.001).

Table 4 shows the relationships between model parameters and self-report metrics on the post-AAC task questionnaire items at 1-year follow-up, and also compares mean scale scores pre-to-post. Notably, all significant correlations found at baseline were replicated at 1-year follow-up. EC again correlated most strongly with self-reported motivations to move toward reward (*r* = − 0.83, *p* < 0.001) and away from negative outcomes (*r* = 0.75, *p* < 0.001). Higher EC also corresponded to higher self-reported anxiety during the task (*r* = 0.30, *p* < 0.001); β again correlated most strongly with self-reported difficulty making decisions on the task (*r* = 0.44, *p* < 0.001) and (reduced) motivations to move toward reward (*r* = − 0.37, *p* < 0.001).

**Table 4:** Post-task self-report questionnaire items at baseline and follow-up, and correlations with computational model parameters at follow-up.

Post-Task Self-Report Questions (Likert Scale: 1 = not at all; 7 = very much)	Mean (SD) Baseline (N = 478)	Mean (SD) 1-Year follow-up (N = 325)	Emotional conflict Paramater (EC)	Decision uncertainty parameter (β)
1. I found the POSITIVE pictures enjoyable	5.04 (1.69)	5.04 (1.54)	.04	− .01
2. The NEGATIVE pictures made me feel anxious Or uncomfortable	4.43 (1.99)	4.25 (1.96)	.30**	.16*
3. I often found it difficult to decide which outcome I wanted	**2.51 (1.73)††**	**2.00 (1.57)††**	− .08	.44**
4. I always tried to move ALL THE WAY TOWARDS the outcome with the LARGEST REWARD POINTS	4.76 (2.30)	4.83 (2.48)	− .83**	− .37**
5. I always tried to move ALL THE WAY AWAY FROM the outcome with the NEGATIVE PICTURE/SOUNDS	2.98 (2.17)	3.17 (2.39)	.75**	.24**
6. When a NEGATIVE picture and sound were displayed, I kept my eyes open and looked at the picture	**5.49 (1.83)†**	**5.17 (2.02)†**	− .47**	− .19**
7. When a NEGATIVE picture and sound were displayed, I tried to think about something unrelated to the picture to distract myself	**2.96 (1.94)††**	**3.34 (2.08)††**	.28**	.09
8. When a NEGATIVE picture and sound were displayed, I tried other strategies to manage emotions triggered by the pictures	**3.26 (1.99)†**	**3.61 (2.07)†**	.24**	.11*

Statistically significent results are highlighted in bold.

^**††**^ = *p* < .01; **†** = *p* < .05 (pre-post differences).

** = *p* < .01; * = *p* < .05 (correlations at follow-up).

### Clinical validity: diagnostic effects

**Figure 3:**
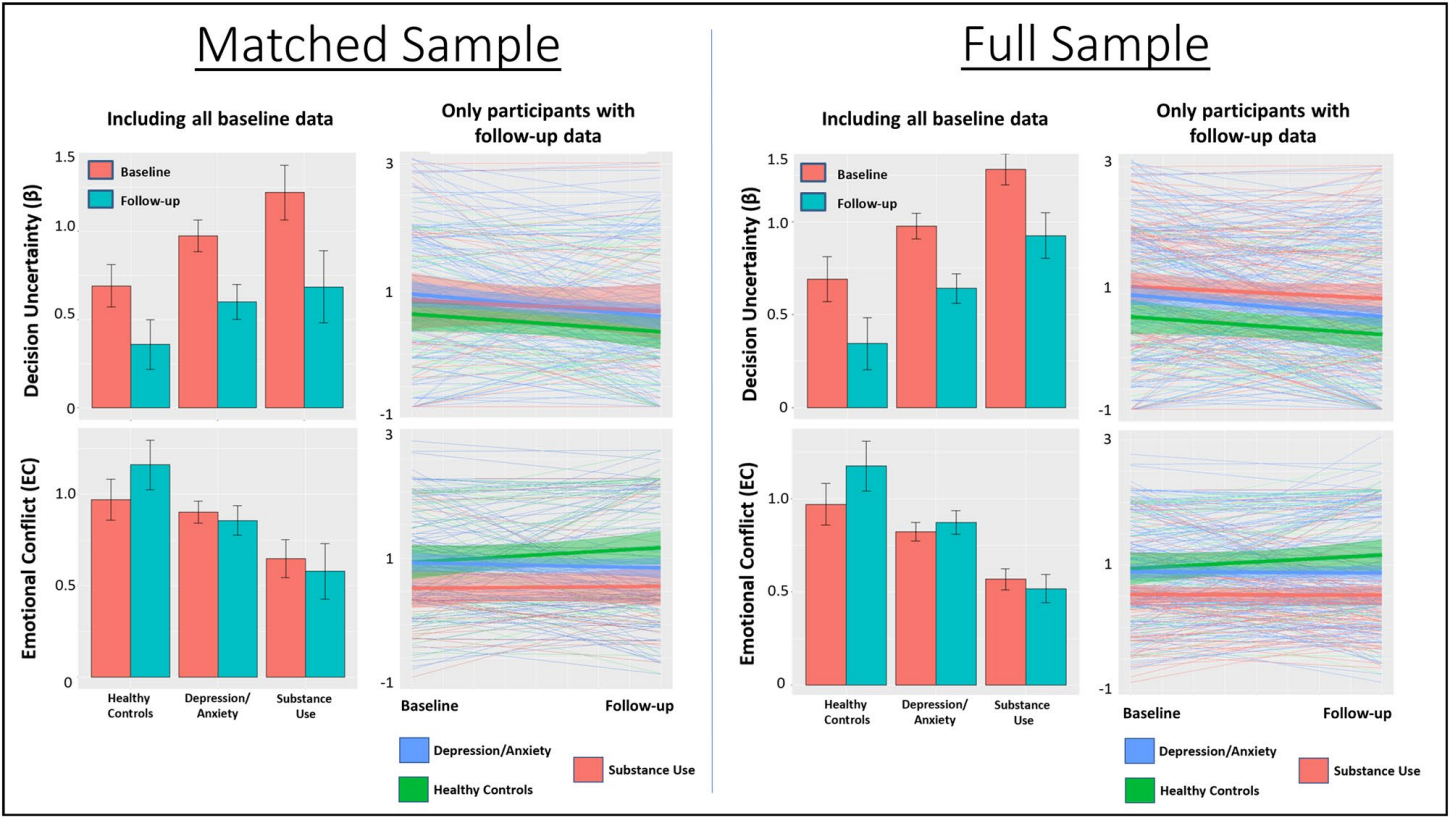
Means and standard errors for model parameters by clinical group and time in both the propensity matched and full samples. Bar graphs include baseline participant values both with and without follow-up data. Spaghetti plots only include participants with both baseline and follow-up data (thick lines indicate group means, surrounding shading indicates standard error). Comparison of bar graphs and spaghetti plots illustrates that relative differences between groups were somewhat more consistent between baseline and follow-up when including all baseline data.

Here, we present group difference results for the matched sample (see Fig. 3). Results in the full sample are provided in Supplementary Materials, and showed a highly similar pattern (as we note more specifically below).

An LME revealed main effects of group (*F*(2, 274) = 3.5,* p* = 0.03) and time (*F*(1, 230) = 14.1,* p* < 0.001) on β values. Post-hoc contrasts revealed that the group main effect reflected higher values in SUDs than in HCs (*p* = 0.009, Cohen’s *d* = 0.29) and the time main effect indexed lower values at 1-year follow up than at baseline (*p* < 0.001); effects of sex, and interactions between group and sex or time were non-significant. A similar pattern was observed in the full sample (see Supplementary Materials).

There was a main effect of group (*F*(2, 275) = 4.6,* p* = 0.01) and sex on EC (*F*(1, 277) = 249.1,* p* < 0.001). Post-hoc contrasts revealed that these effects reflected higher EC in females and greater EC in HCs than in both DEP/ANX (*p* = 0.02, *d* = 0.21) and SUDs (*p* = 0.004, *d* = 0.63). A similar pattern was observed in the full sample (see Supplementary Materials); however, in the full sample there was also a group by sex interaction suggesting that group effects were driven by females.

### Clinical prediction

When accounting for baseline scores, age, sex, and WRAT, exploratory linear regressions (excluding HCs, full sample) revealed a significant relationship between EC at baseline and change in BIS scores from baseline to follow-up (*t*(6, 242) = 2.03, *p* = 0.04), indicating that higher EC values at baseline predicted larger increases (or smaller decreases) in behavioral inhibition over time. For descriptive statistics and group difference analyses for BIS scores (motivated by these results), see Supplementary Materials. No other effects were found supporting the ability of baseline parameter values to predict changes over time in PHQ, OASIS, DAST, BAS, ASI, PROMIS depression/anxiety scales, or PANAS positive and negative affect scales (all *p*s > 0.05). Additional exploratory analyses also did not find any significant associations between pre-post changes in parameter estimates and pre-post changes in any of these measures.

### Descriptive analyses

To help interpret computational modelling results and allow comparability with previous ACC studies, we also performed secondary LMEs on traditional behavioral task variables. These analyses and descriptive statistics for task-related self-report and traditional performance variables (RT, approach behavior) are provided in Supplementary Materials. Among other effects, these revealed that RTs were faster in HCs than both clinical groups in the AV condition. HCs showed greater avoidance than the clinical groups for all but the APP trials. Greater within-subject choice variability was seen in SUDs than in the other groups. Both patient groups reported greater decision difficulty than HCs, and HCs reported less approach motivation and greater avoidance motivation than the patient groups.

### Short-term test–retest reliability

Descriptive statistics for behavior in the second sample of healthy participants, with a test–retest interval of approximately 2–3 weeks apart, are shown in Supplementary Materials. Analysis of this data showed excellent reliability for the EC parameter (ICC = 0.84 (*F*(29, 29) = 11.3,* p* < 0.001) and fair reliability for the β parameter (ICC = 0.54; *F*(29, 29) = 3.32,* p* < 0.001).

## Discussion

In this paper, we have demonstrated that a computational modelling approach to studying AAC task behavior is able to capture moderately stable individual differences—and stable psychiatric group differences—over a period of 1 year between a baseline and follow-up visit. Despite the lengthy one year time period between visits, ICCs were moderate. At this 1-year follow-up, relationships between model parameter estimates and task-related RTs, self-reported motivations, and descriptive behavioral measures were consistent with what we previously found at baseline. Of particular interest, greater decision uncertainty (β) in the model remained associated with greater self-reported decision difficulty and slower RTs, while greater emotion conflict (EC) in the model remained associated with greater self-reported anxiety and avoidance motivation. Further, differences between diagnostic groups in model parameters were also stable between baseline and 1-year follow-up. Namely, the SUD group showed greater β values than HCs at both timepoints (with DEP/ANX taking intermediate values between the two), and both patient groups showed lower EC (see Fig. 3).

When reliability was assessed in a second healthy sample with a shorter test–retest interval (approximately 2–3 weeks), ICCs were excellent for EC but remained fair for β. This suggests that the lower ICCs for EC over the 1-year period in the main sample may reflect true underlying changes in participants’ decision processes (as opposed to measurement error). This parameter therefore shows promise as a repeatable assessment tool at the individual level. In contrast, results for β may suggest greater measurement error at the individual level. This may also be due in part to task familiarity effects, in which participants become more confident in a decision strategy with repeated task performance (i.e., consistent with the reduced mean and variability in β values at retest in this second sample; see Supplementary Materials). These findings complement previous work reporting fair to excellent reliability for descriptive behavioral measures and for activation in some brain regions of interest to the same AAC task during functional MRI (e.g., dorsal anterior cingulate during decision-making; amygdala during processing of affective outcomes;^58^).

Together, these results (1) support the validity of the model/task in being able to provide moderately consistent results with repeated use over a relatively long period of time, and (2) support the ability of model parameters to act as stable markers of clinical group differences. The moderate ICCs suggest relatively consistent individual differences (accounting for the stable group differences) that may have also been influenced by heterogeneous experiences over the 1-year period. However, we were unable to identify other variables that could account for variability in pre-post changes (e.g., no relationships with symptom changes).

The diagnostic group differences increased confidence in the somewhat unexpected findings in our previous report^20^. Specifically, the finding that HCs show greater EC values than the patient groups appears reliable. This is consistent with HCs exhibiting greater avoidance during CONF and AV conditions, and greater approach during APP conditions (i.e., in the full sample) in descriptive analyses (see Supplementary Materials). It is also consistent with the findings that HCs exhibited faster RTs in the AV condition and greater self-reported avoidance (and reduced approach) motivations during the task (i.e., marginal in the matched sample, but significant in the full sample; see Supplementary Materials). In other words, HCs exhibited behavior consistently driven by the potential affective outcomes in expected directions (e.g., avoiding more in response to negative outcomes; approaching more when there was only reward), whereas the behavior of the DEP/ANX and SUD groups was less expected. This could be related to reduced reward-seeking in depression, which is consistent with our observation that those with lower EC values at baseline tended to have reduced levels of behavioral inhibition (BIS scores) over time. Lower EC (and less avoidance) in patients might also suggest a decreased sensitivity or reactivity to negative stimuli (consistent with a previous body of work in SUDs; see^59–63^). One additional possibility is that the patients’ experience of greater ambiguity or uncertainty when judging the appropriate response under different conflict conditions may lead them to place the avatar near the middle of the runway rather than fully committing to an approach or avoidance strategy (as HCs tended to do; see histograms within Supplementary Materials).

Our results also lend added confidence to our prior result demonstrating greater β values in SUDs. This was part of a larger consistent pattern involving greater self-reported decision difficulty in SUDs (and DEP/ANX) than HCs, and slower RTs in the AV condition (i.e., suggesting less confidence in what choice to make; see Supplementary Materials). They also provide added support for the possibility that elevated decision uncertainty during AAC may be an important aspect of psychopathology, which previous behavioral analyses have not distinguished from emotional conflict^10,64^. Notably, there were stable reductions in β values for all three groups. Due to the generality of this effect across all participants, it is most plausibly an effect of task familiarity at the 1-year follow-up, as participants may have become more confident in their decision strategy (as also mentioned above with respect to the smaller test–retest sample).

Our exploratory finding that EC values at baseline could predict behavioral inhibition at follow-up suggests the possibility that this measure could have predictive clinical utility. However, this will need to be confirmed in an independent sample. Dissapointingly, we did not find evidence that parameter estimates at baseline could predict changes in other dimensional or symptom measures over the following year, suggesting that our paradigm may be better thought of as providing a stable marker of membership to one or more diagnostic categories. However, predictive utility in this sample may have been limited due to the heterogeneity in experiences over the 1-year period (e.g., with some participants seeking various forms of treatment while others did not). It may still be beneficial to determine whether these AAC parameters could be useful for predicting outcomes of specific pharmacological or behavioral treatments targeting threat or reward responsivity and/or decision-making.

## Limitations and conclusions

While our findings offer strong support for the validity of the model and its ability to provide moderately stable individual difference estimates in clinical populations with repeated testing, it is important to highlight persisting limitations. First, while the group differences were remarkably stable, ICCs were only moderate, and the factors leading to pre-post differences remain to be identified. Results in the small follow-up sample of healthy participants indicated that the EC parameter had excellent reliability when retested after a shorter period of 2–3 weeks, which suggested that changes in this parameter over the 1-year period could reflect genuine changes in underlying decision processes. However, an important question to be addressed in future research is whether ICCs are also higher in patient populations when the task is repeated over shorter intervals of time, and whether larger samples would confirm the effect in healthy subjects. If so, this would provide stronger support for the use of these model parameters as stable individual difference measures, whereas our lengthy 1-year follow-up period may have involved meaningful changes in underlying mechanisms of interest. It is also worth highlighting that ICCs could be limited by parameter estimation itself. Specifically, while simulations indicated strong recoverability, any variability in estimation accuracy between baseline and follow-up would reduce ICCs. Next, while our results replicate previous effects observed in the same sample, they do not replicate our initial results in a new sample of participants. Replication in a new sample will be necessary to support the ability of the task/model to highlight differences that generalize to broader psychiatric populations. The second 500 participants of the T1000 are set aside specifically for pre-registered replication analyses and we plan to next replicate these results in this confirmatory sample.

Another limitation stems from the fact that 32% of participants did not complete the follow-up visit. We can therefore not rule out that selection effects contributed to our results. While supplementary analyses did not reveal differences in baseline depression/anxiety symptoms between completers and non-completers, those who did not complete the follow-up visit did have higher substance use severity at baseline (i.e., corresponding to the fact that a smaller proportion of individuals in the SUDs group [53%] returned for the follow-up visit relative to the other groups; note that these differences in substance use severity between completers and non-completers were not present when restricting analyses to the SUDs group alone). A final limitation concerns our inability to match the proportion of males and females across groups, where there were a greater number of females in the DEP/ANX group than the other two groups. As in our previous study on the baseline data, females also had greater EC values. This allows for the possibility that greater EC values in the DEP/ANX group could be explained in part by including a greater number of females. Although not the focus of this study (as this effect was stable over time), this sex difference may relate to other work showing that reduced reward motivation plays a larger role in avoidance behavior in females (12)—raising the possibility that different targets of intervention may be most effective for females versus males or depending on the relative role of reward and avoidance motivations.

With these limitations in mind, our results demonstrate that AAC behavior shows consistent patterns of individual and group differences over a 1-year period and that the present task and model may act as a stable and reliable marker of these differences over repeated performance—a necessary feature for many practical uses in computational psychiatry. Future research should replicate these findings in a new sample and further investigate their potential clinical relevance.

## Supplementary Information

Supplementary Information 1.

Supplementary Information 2.

## Data Availability

The data used in the analyses reported in this study and the code for running the AAC paradigm are available upon request. The Matlab code used to build the task model and estimate parameters is included in Supplementary Materials.
