# Supplementary material for: Long-term stability of computational parameters during approach-avoidance conflict in a transdiagnostic psychiatric patient sample
Source: Sci Rep. 2021 Jun 3;11:11783. doi: 10.1038/s41598-021-91308-x (PMC8175390; doi:10.1038/s41598-021-91308-x)
Supplement: Supplementary file 1 — Supplementary Information 1. [file 41598_2021_91308_MOESM1_ESM.doc]

**Supplementary Materials:**

**Long-term stability of computational parameters during approach-avoidance conflict in a transdiagnostic psychiatric patient sample**

Ryan Smith PhD, Namik Kirlic PhD, Jennifer L. Stewart PhD, James Touthang BS, Rayus Kuplicki PhD, Timothy J. McDermott MA, Samuel Taylor BS, Sahib Khalsa MD, PhD, Martin P. Paulus MD, Robin Aupperle PhD

Laureate Institute for Brain Research, Tulsa, OK, USA

**Additional methods information**

**Approach-avoidance conflict (AAC) task**

The AAC task used here is identical to that used in our previous paper (1). Prior to performing the task, participants received detailed instructions (also provided as an appendix in our previous paper) and completed four practice trials to ensure sufficient understanding. On each trial, a runway was shown with a picture of an avatar in a starting position above the runway. Pictures were also shown on each side of the runway, indicating the types of stimuli that could be presented at the end of the trial (see **Figure 1** within the main text). Specifically, a sun or cloud represented potential positive or negative affective stimuli, respectively; while the height of red fill in a rectangle signified the number of points that would be received in conjunction. On each trial, participants could press the left or right arrow keys to move the avatar from its starting position to any other position (9 possible locations) on the runway, and they were asked to choose one ending position on each trial. They were told that each ending position corresponded to a specific probability of observing different stimuli at the end of the trial. These stimuli included a positive or negative affective image-sound combination (indicated by the sun or cloud, respectively), and a certain level of reward points (indicated by the height of red fill in the rectangle). The ending position of the avatar determined the probability of each of these outcomes occurring. Before starting the task, participants were told the specific probabilities of observing each stimulus for each runway position, and that these probabilities were stable across the task. Thus, there was no learning in this task, and no measure of better/worse performance; participants simply indicated their preferred location on the runway (based on the probabilities of each outcome) on each trial. The probabilities told to the participants were based on the distance from each stimulus (e.g., being closer to the sun image indicated a higher probability of observing the positive stimulus). From left to right on the runway, the probabilities = [.9/.1, .8/.2, .7/.3 .6/.4, .5/.5, .4/.6, .3/.7, .2/.8, .1/.9]. The starting position of the avatar (middle, left end, or right end) was counterbalanced across trials (for each trial type; see below) to control for its potential influence on the participants’ choice.

The affective image-sound combinations were gathered from the International Affective Picture System [IAPS] (2), International Affective Digitized Sounds [IADS] (3), and other freely available audio files (see further description in previous reports using this task; (4, 5)). The “reward” included 0, 2, 4, or 6 points presented along with a trumpet sound. The specific task instructions and stimuli used for the AAC task are provided as an appendix in our original paper (1). There were five trial types (see **Figure 1** within the main text), which were indicated to participants by the images shown on each side of the runway on each trial. Each trial type was named in reference to the behavioral motivation presumably elicited by the negative/positive affective stimuli and/or the reward points: (1) ‘Avoid-threat’ (AV), in which 0 points were offered for both possible stimulus outcomes and thus, the only explicit motivation was to avoid the negative affective stimulus. (2) ‘Approach-reward’ (APP), in which 2 versus 0 points were offered, each with positive affective stimuli. For this condition, the only explicit motivation was to approach the rewarded outcome. (3)-(5) Three levels of ‘Conflict’ in which the negative affective stimulus was presented in addition to winning either 2 (CONF2), 4 (CONF4), or 6 (CONF6) points, while 0 points were offered for the other possible outcome, in which a positive affective stimulus would be presented. The task consisted of a total of 60 trials, with 12 of each of the five trial types. After task completion, a screen appeared displaying total points received and an award ribbon. As in previous administrations of the task (4, 6), points did not correspond to monetary reward. Notably, previous research has shown that paradigms involving either non-monetary or monetary reward elicit similar neural activation patterns in reward-sensitive brain regions (7, 8), which could suggest similar motivational influences. Behavioral variables consisted of both chosen avatar position and response times (RTs; i.e., time to initial button press) during each trial. Participants were also asked to fill out the same post-task Likert scale questionnaire as in our previous study, which asked about their experience during the task (these questions are listed in **Table 4** within the results section of the main manuscript).

**Computational model description**

To model behavior on the AAC task described above, we adopted a Markov decision process model under the active inference framework (9-11). This approach requires writing down a generative model comprised of a few specific variables (that can each take a number of discrete values) and matrices describing the probabilistic relationships between those variables. The first two sets of variables are observations (*o*) and hidden states (*s*), where the relationships between these variables at a time (*t*) are described by a set of matrices referred to as **A** matrices – which encode the way that hidden states generate observations,
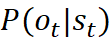
. The probability that one hidden state will transition into another hidden state over time,
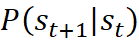
, is encoded by a set of transition matrices referred to as **B** matrices (i.e., where these transitions also depend on selected actions, as described below). The degree to which an individual prefers (values) some observations over others is encoded within a matrix referred to as the **C** matrix, which, as explained further below, is technically modeled as a fixed set of prior expectations over observations,
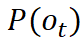
. Finally, a set of matrices referred to as **D** matrices encode the probability of starting out (e.g., at the beginning of each trial) in one hidden state vs. another,
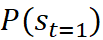
.

Belief updating in this class of models makes use of a set of variational update equations that approximate Bayesian inference. In these equations **D** is used as a prior for expected states (under each allowable sequence of actions or policy π) at the first time step (*t* = 1), while **B** provides priors over states at subsequent time steps in a trial, and these priors are integrated with the evidence that observations provide in favor of states, as specified by **A**:


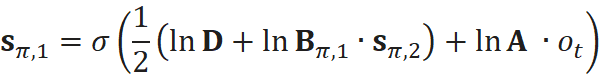


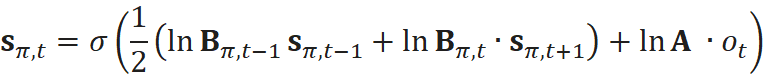


Please note that when the dot (
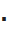
) notation is applied to matrices in these and other model equations, this indicates transposed matrix multiplication. Also note that the time index *t* refers to time points *about* which an individual holds beliefs, where beliefs about each time point can be updated after each new observation. For example, before an observation an individual might have a (often uncertain) belief about the state (and therefore outcomes) at *t* = 2, and will update their beliefs about the state at *t* = 2 after a new observation. In the active inference literature these beliefs *about* time points are often instead denoted with the Greek letter tau (τ) in order to distinguish them from the times (*t*) *at* which new observations are presented (for details, see (12)).

To implement decision-making in this class of models requires that one write down the set of allowable policies that can be selected by the model. Technically, each action available at a given time point corresponds to a particular transition matrix (i.e., one of several **B** matrices) that defines a state transition under the control of the simulated individual (the “agent”) described by the model,
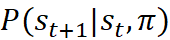
, such that each allowable policy corresponds to the selection of a sequence of state transitions. The impact of the agent’s model on policy selection is also regulated by an expected precision term (
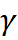
) that can be thought of as encoding the agent’s confidence in its action model – that is, confidence in the ability to select the best action based on current beliefs. When expected precision is high, policy selection is strongly determined by a posterior distribution over policies (i.e., a distribution specifying inferred policy values, described further below); when expected precision is low, this attenuates how sensitive an agent is to differences in the value of different policies during decision-making, and tends to promote behavior that appears more inconsistent, as it corresponds to higher uncertainty in decision-making. The model includes a prior over expected precision (β), which is formally the ‘rate’ parameter for a standard Gamma prior over the expected precision (
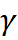
). That is:
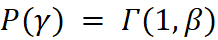
.

The probability of a policy being selected is in turn determined by the observations it is expected to lead to and how much they diverge from preferred observations (the prior preference distribution defined by the **C** matrix). Posterior policy probabilities can be inferred based on the following equation:


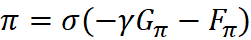


Here *F* and *G* refer to the free energy and expected free energy of a policy (respectively), where lower values of each promote selection of the corresponding policy. *F* can be thought of as encoding the accuracy of updated model predictions, while also taking into account how much prior beliefs need to be revised to reach high predictive accuracy. *G* is a measure of the divergence between actual and preferred observations, while also taking into how much a policy is expected to reduce uncertainty about states:


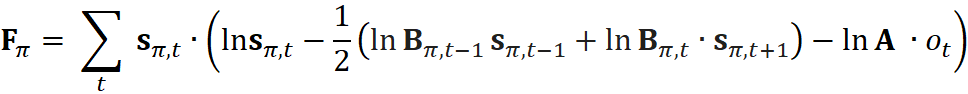


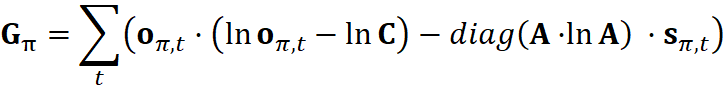


Policies with higher probability will therefore better minimize the divergence between actual and preferred observations while also minimizing uncertainty. Actions are then chosen by sampling from the resulting posterior distribution over policies.

In the active inference scheme, approximately Bayes optimal inference is performed using standard variational message passing algorithms that minimize variational free energy, which, as described above, is an approximate means of minimizing the divergence between expected and preferred observations (13, 14). For a more detailed description and derivation of the associated belief update equations, see (9, 10, 15); here we implement these updates using the spm_MDP_VB_X.m routine freely available within the DEM toolbox implemented within the SPM12 software package (Wellcome Trust Centre for Neuroimaging, London, UK, <http://www.fil.ion.ucl.ac.uk/spm>).

To model the AAC task, one must therefore write down the sets of observations, hidden states, policies, and associated matrices that are sufficient to generate an individual’s behavior during the task. Here, we included three categories of observations, corresponding to the observed position on the runway (10 possible observations, corresponding to a “starting” position and each of the nine positions on the runway that could be chosen), the task condition (five possible observations, corresponding to the five trial types), and the stimulus observed at the end of each trial (seven possible observations: a “starting” observation, negative affective stimulus + 0 points, positive affective stimulus + 0 points, positive affective stimulus + 2 points, negative affective stimulus + 2 points, negative affective stimulus + 4 points, and negative affective stimulus + 6 points). We included two categories of hidden states, corresponding to the individual’s beliefs about their position on the runway and beliefs about the trial type. The mappings (**A** matrices) from beliefs about runway position and beliefs about trial type to observed positions and observed trial types (respectively) were specified as identity matrices, such that there was no uncertainty about the trial type or runway position. The **A** matrix specifying the mapping from beliefs about runway positions (columns in the matrix presented below) to observable stimuli (rows in the matrix presented below) defined the probabilities of observing each possible stimulus combination conditional on both the runway position and the trial type. For example, under the “avoid threat” (AV) trial type, the mapping from the runway positions to the different stimuli was as follows (columns = states, rows = observations):


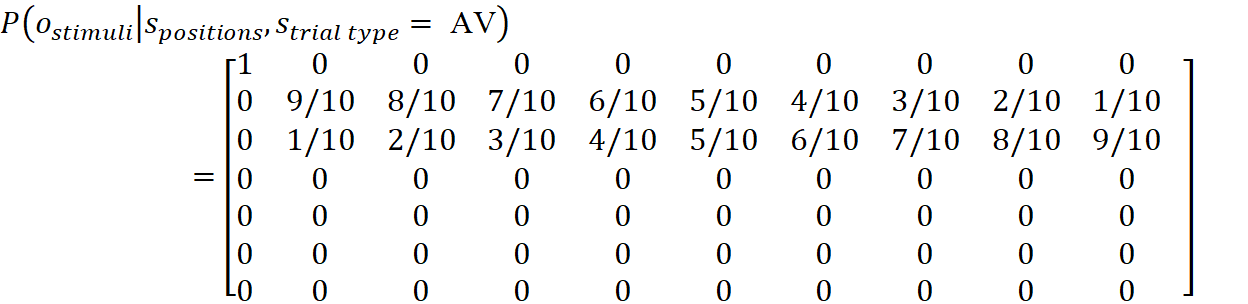


Rows from top to bottom correspond to: starting observation, negative affective stimulus + 0 points, positive affective stimulus + 0 points, positive affective stimulus + 2 points, negative affective stimulus + 2 points, negative affective stimulus + 4 points, negative affective stimulus + 6 points. Thus, this matrix says that, under the AV trial type, the closer one was to the left side of the runway the higher the probability of observing the negative affective stimulus + 0 points, whereas the opposite mapping was true for observing the positive affective stimulus + 0 points. It also says that, under the AV trial type, the probability of observing all other possible stimulus combinations was 0. Analogous matrices defined the probabilities associated with each of the other trial types, which are depicted in **Figure S1**.


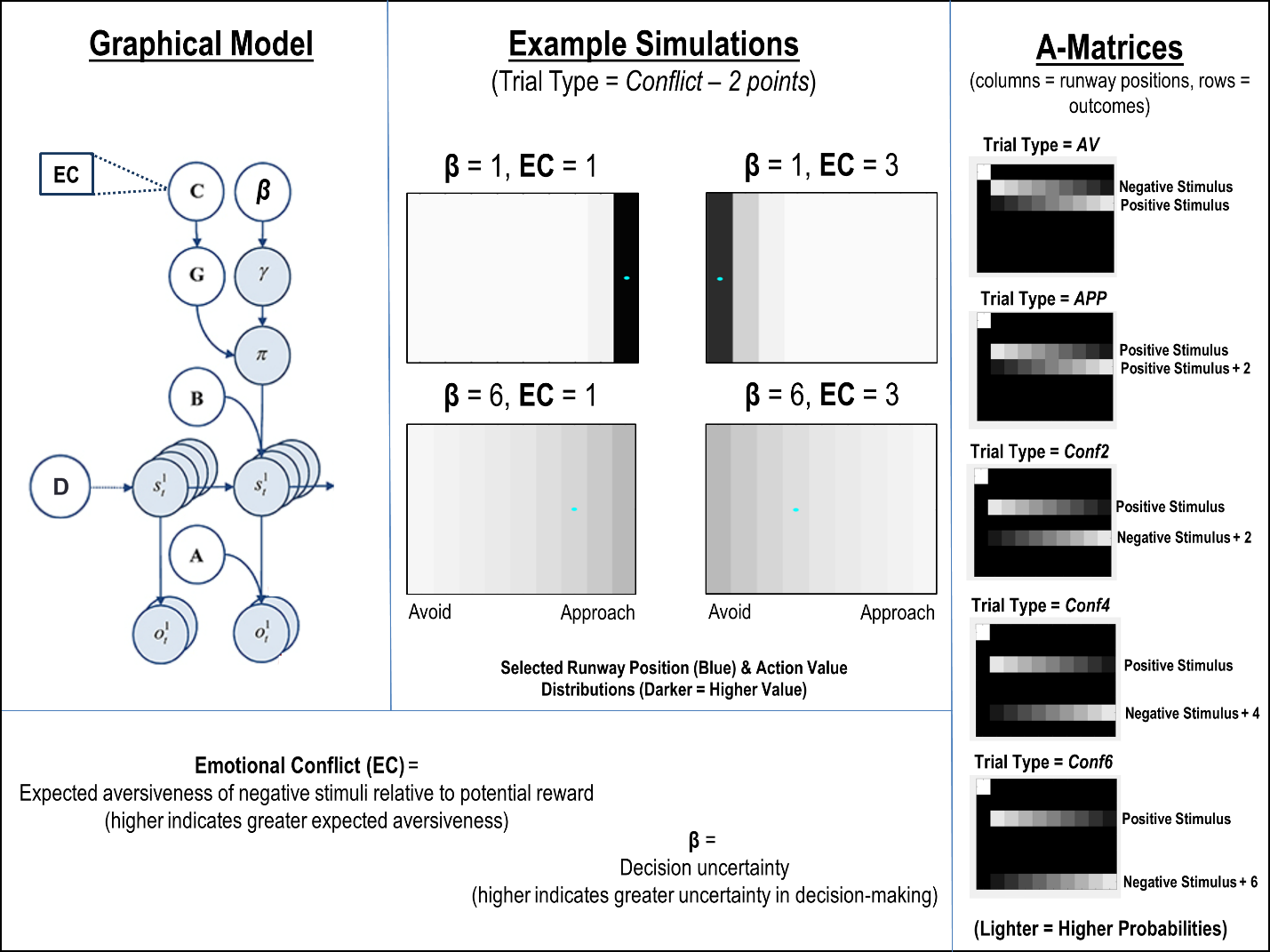


**Figure S1**. Computational Model. (Top Left) Depicts the Markov decision process used to model the approach-avoidance conflict task. The generative model is depicted graphically, such that arrows indicate dependencies between variables. Observations (o) depend on hidden states (s), where this relationship is specified by the **A** matrix, and those states depend on both previous states (as specified by the **B** matrix, or the initial states specified by the **D** matrix) and the sequences of actions/policies (π) selected by the agent. The probability of selecting a particular policy in turn depends on the expected free energy (**G**) of each policy with respect to the prior preferences (**C**) of the agent. The degree to which expected free energy influences policy selection is also modulated by an expected precision term (**γ**), which is in turn dependent on a prior policy precision parameter (β) – where higher values of β promote greter decision uncertainty (i.e., less influence of the differences in expected free energy across policies). For more details regarding the associated mathematics, see (10, 16). In our model, the observations were cues indicating the trial type, cues indicating the position of the avatar, and the outcome stimuli (**A**-Matrices in right panel;columns = start state + runway positions, rows = outcome stimuli, lighter = higher probability). The hidden states included beliefs about trial type and avatar position, and the policies included the choice to move the avatar to any other position on the runway. (Bottom Left) The model parameters corresponded to the degree to which the negative stimulus was dyspreferred relative to the degree to which the points were preferred within the **C** matrix (“emotional conflict”; EC), as well as the prior policy precision parameter β reflecting decision uncertainty. (Top Middle) Example simulations of action selection under different parameter values during the “Conflict + 2 Points” trial type. Blue dots indicate chosen actions and darker colors indicate higher action values in the model (i.e., columns 2-10 correspond to choosing each of the 9 runway positions; right = negative stimulus + 2 points, left = positive stimulus; row 1 and column 1 correspond to the “start” state and associated observation). Trial Types: AV = Avoid; APP = Approach; CONF2, CONF4, and CONF6 indicate Conflict + 2 Points, 4 Points, or 6 Points, respectively.

The hidden states corresponding to runway positions were under the control of the agent, such that there was one **B** matrix encoding a transition from the starting state to each of the nine positions, corresponding to nine allowable one-step policies. The single **B** matrix that was specified for the other hidden state category (corresponding to trial types) was an identity matrix, encoding the belief that trial type remained stable within each trial. The **C** matrix was specified such that the value assigned to each possible stimulus observation was determined by three parameters corresponding to the subjective value of the positive affective stimulus, the subjective value of the negative affective stimulus, and the subjective value assigned to each point that could be won during the task. Here, we chose to fix the value of the positive affective stimulus at an “anchor” value of
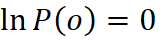
 and set the value of each point to
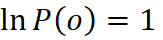
 (i.e.,
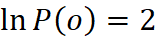
when winning 2 points, etc.). In other words, we quantified subjective value in terms of natural units – associating each point with a natural unit. This was motivated by the fact that the number of points ranged from 0 to 6. This covers the natural range of prior preferences, when interpreted in terms of log probabilities. We then estimated the value (subjective aversiveness) of the negative affective stimulus. This parameter indicated the “emotional conflict” (EC) – that is, the relative expected aversiveness of the affective stimuli relative to the expected (subjective) reward value assigned to each point. The *t* = 2 column in the **C** matrix specifying preferences over outcomes was therefore as follows:


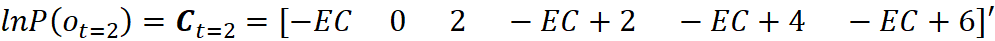


From left to right, this assigns value to observting the negative stimulus, the positive stimulus, the positive stimulus + 2 points, the negative stimulus + 2 points, the negative stimulus + 4 points, and the negative stimulus + 6 points.

The **D** matrix for the hidden state category corresponding to runway positions assigned a probability of 1 to starting within the starting state at the beginning of each trial and 0 otherwise, [1 0 0 0 0 0 0 0 0 0]', whereas the **D** matrix for the hidden state category corresponding to trial types assigned equal probability to each trial type, [.2 .2 .2 .2 .2]', reflecting the belief that no trial type was more likely to occur than any other. As mentioned above, prior policy precision (β) was not fixed in advance, but was also estimated for each individual.[[1]](#footnote-2)

Our computational phenotyping approach used Bayesian inference at two levels (17). First, each participant’s responses were modeled under ideal Bayesian assumptions – using the MDP formulation of choice behavior described previously. We then used Variational Bayes to estimate each participant’s prior beliefs that maximized the likelihood of their responses, as described in (18). In other words, the observation model for estimating subject-specific preferences and precision was based on the assumption that subjects were using (active) Bayesian inference. In this setting, active inference can be seen as a generalization of Bayesian decision theory that replaces the expected value or utility with expected log evidence or marginal likelihood for a generative model of the task at hand (18). Technically, this means that subjective responses are sampled from their posterior beliefs about the best course of action, where these posterior beliefs depend upon their prior preferences about the consequences of a decision – and the information gain afforded by their actions. This posterior distribution over behavioral responses can then be used to assess the likelihood of responses under different prior beliefs. We optimized these preferences (and precision of posterior beliefs about policies) using this likelihood and standard variational Laplace (19).

This estimation approach has the advantage of preventing overfitting, due to the greater cost it assigns to moving parameters farther from their prior values. In this case, we chose to estimate two parameters (EC, β), which required setting prior means and prior variances for each parameter. The prior variance was set to a high precision value of 2^-2^ for each parameter (i.e., deterring overfitting), and the prior means (specified as lognormal priors) were set as follows: EC = 2 (made negative in the model) and β = 1. While other prior values could have been chosen, our decision for selecting these priors was motivated in part by initial simulations confirming that parameter values were recoverable under these prior values (described below). In addition, by selecting a prior value of (-)2 for EC, this also entailed that the task condition in which participants could observe the negative affective stimulus and receive the lowest number of (two) points would correspond to maximum conflict (i.e., these values would sum to 0). β = 1 is also a standard rate parameter often used for gamma priors.

In our previous paper, we also considered two other models: a simpler 1-parameter model including no decision uncertainty term (only estimating EC), and a more complex 3-parameter model that fit the subjective value of the points as well. Formally, the simpler model simply removed the expected policy precision term (
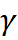
), such that posteriors over policies were simply:


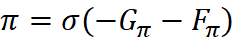


The more complex model formally adjusted the **C** matrix displayed above to include a parameter that scaled the value of each points (Pval) as follows:


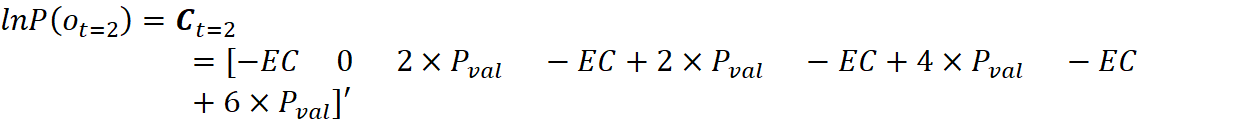


We first assessed whether model parameters were recoverable within simulated data, while varying true parameter values and prior values during model estimation. We then assessed whether posterior parameter estimates reliably approached the true parameter values specified in the simulations. Parameter estimates for the 3-parameter model did not appear recoverable, and were dependent on prior values, due to the fact that only the relative value of the negative stimuli vs. points ultimately influenced behavior. Therefore, we did not use this model. In contrast, estimates from the simpler 1-parameter model did appear recoverable, as they reliably approached true values from different starting priors. However, Bayesian model comparison (based on (20, 21)) showed that this model performed worse than the 2-parameter model (protected exceedance probability = 1). Parameters were recoverable for the 2-parameter model, as indicated by simulations confirming that estimated parameter values approached true parameter values across 46 representative combinations present in participant data. Specifically, the Pearson correlation between true and estimated decision uncertainty values in these simulations was *r* = .94, and that for EC values was *r* = .9. We therefore selected this model for our further analyses.

**Supplementary Results**

**Intraclass correlations in model parameters by group**

ICCs for β by group were as follows:

**HCs**: ICC = .38 (*F*(47,47) = 2.2, *p* = .004)

**DEP/ANX**: ICC = .47 (*F*(191,191) = 2.8, *p* < .001)

**SUDs**: ICC = .41 (*F*(83,83) = 2.4, *p* < .001)

ICCs for EC by group were as follows:

**HCs**: ICC = .60 (*F*(47,47) = 4.1, *p* < .001)

**DEP/ANX**: ICC = .48 (*F*(191,191) = 2.9, *p* < .001)

**SUDs**: ICC = .48 (*F*(83,83) = 2.9, *p* < .001)

**Intraclass correlations in descriptive task measures**

Across participants, ICCs also showed fair to good reliability in each of the five task conditions for both RTs (ICCs between 0.5 and 0.61) and mean chosen runway position (ICCs between 0.4 and 0.56). Similar results were found when examining groups separately. For RTs, HCs: ICCs between 0.44 and 0.57; DEP/ANX: ICCs between 0.5 and 0.62; SUDs: ICCs between 0.52 and 0.63. For mean chosen runway position, reliability differed by group and condition. In HCs, the three conflict conditions showed fair to good reliability (ICCs between 0.58 and 0.62), while reliability in the AV and APP conditions was poor (ICC = 0.23 and -.03, respectively). In contrast, mean chosen runway position in both patient groups showed fair to good reliability in the AV and APP conditions (DEP/ANX: ICCs = 0.63 and 0.47, respectively; SUDs: ICCs = 0.45 and 0.44, respectively), and fair reliability in the three conflict conditions (DEP/ANX: ICCs between 0.41 and 0.44; SUDs: ICCs between 0.42 and 0.51).

**Intraclass correlations in clinical measures**

From baseline to 1-year follow-up, dimensional measures showed the following single-measure consistency intraclass correlations [ICC(3, 1)]:

**PHQ**: ICC = .54; *F*(246,246) = 3.39, *p* < .001

**OASIS**: ICC = .61; *F*(247,247) = 4.07, *p* < .001

**DAST**: ICC = .43; *F*(246,246) = 2.50, *p* < .001

**ASI**: ICC = .70; *F*(295,295) = 5.69, *p* < .001

**BIS**: ICC = .68; *F*(320,320) = 5.32, *p* < .001

**BAS-Reward**: ICC = .61; *F*(320,320) = 4.18, *p* < .001

**BAS-Fun Seeking**: ICC = .64; *F*(320,320) = 4.61, *p* < .001

**BAS-Drive**: ICC = .70; *F*(320,320) = 5.69, *p* < .001

**PROMIS-anxiety:** ICC = .56; *F*(317,317) = 3.52, *p* < .001

**PROMIS-depression**: ICC = .62; *F*(316,316) = 4.20, *p* < .001

**PANASX-positive affect**: ICC = .60; *F*(320,320) = 4.0, *p* < .001

**PANASX-negative affect**: ICC = .62; *F*(320,320) = 4.32, *p* < .001

These ICCs generally indicated that these measures had fair to good reliability over the 1-year period.

**Comparison of symptoms in participants who did and did not complete the follow-up visit**

In both the matched and full samples, two-sample t-tests did not show significant differences in depression or anxiety scores (PHQ and OASIS) at baseline in those who did vs. did not return for the follow-up visit. Those who completed the follow-up visit did have significantly lower substance use symptom severity (DAST) at baseline than those who did not (matched sample: *t*(95) = 3.61, *p* < .001; full sample: *t*(271) = 4.95, *p* < .001).

**Relationship between model parameters and demographic variables in the full sample at follow-up**

The EC parameter was negatively correlated with age scores (*r* = -.11, *p* = .04), did not differ by sex, and was positively correlated with WRAT scores (*r* = .19, *p* < .001). The β parameter was positively correlated with age (*r* = .14, *p* = .01), did not differ by sex, and negatively correlated with WRAT scores (*r* = -.25, *p* < .001).

**Model parameter group difference analyses in the full sample**

Here we present group difference results for the full sample (see **Figure 3**, main text), based on identical LMEs including sex, group, and their interactions with session.

An LME revealed main effects of group (*F*(2,472) = 10.75, *p* < .001) and time (*F*(1,377) = 19.01, *p* < .001) on β values. Post-hoc contrasts revealed that these effects reflected higher values in SUDs than in HCs (*p* < .001, Cohen’s *d* = .60) and DEP/ANX (*p* < .001, *d* = .22) and lower values at 1-year follow up than at baseline (*p* < .001); effects of sex, and interactions between group and sex or time were non-significant.

There was a main effect of group (*F*(2,473) = 11.17, *p* < .001) and sex (*F*(1,451) = 9.61, *p* = .002) on EC, as well as a group by sex interaction (*F*(2,459) = 4.70, *p* = .01). Post-hoc contrasts revealed that the main effects reflected higher EC in females (*p* = .002) and greater EC in HCs than in both DEP/ANX (*p* = .007, *d* = .23) and SUDs (*p* < .001, *d* = .76), as well as greater in DEP/ANX than SUDs (*p* = .007, *d* = .52). The interaction reflected significant differences between all groups in females (*p* < .001 for each), but no significant differences between groups in males (*p*s between .24 and .51). There was no effect of time or interaction between group and time.

**Standard descriptive analyses in the matched sample**

Descriptive statistics for task-related self-report and traditional performance variables (RT, approach behavior) are provided in in **Table S1** and **Table S2**; for histograms of approach-avoidance behavior by group and condition, see **Figure S2**. In identical LMEs to those performed above, average RTs across trial types were significantly faster in the 1-year follow-up compared to baseline (F(1,216) = 8.54, p = .004); effects of sex and group (as well as their interaction with time) were non-significant. When analyzing separately by condition, this same pattern was observed in the three conflict conditions. However, the two non-conflict conditions showed a different pattern. The AV condition showed a main effect of group (F(2,275) = 5.30, p = .006), but no effect of time or other model terms. The main effect of group reflected faster RTs in HCs than in SUDs (p = .002) and DEP/ANX (p = .01). The APP condition showed no significant effects, although we note marginal results for group and time (p = .056 and .054, respectively), suggesting faster RTs in HCs than SUDs (p = .02) and faster RTs at 1-year follow-up (p = .054). We note that this effect of group was significant in the full sample (p = .005), suggesting that this marginal effect reflected reduced power. Other results in the full sample showed a similar pattern as reported here (see below).

In identical LMEs to those preformed above, average chosen runway position across the three conflict conditions showed greater approach behavior in males than females (F(1,269) = 7.46, p = .007). There was also an effect of group (F(2,275) = 2,72, p = .04), reflecting less approach behavior in HCs than in DEP/ANX and SUDs (p = .02 and .03, respectively). The same pattern was also found when analyzing the three conflict conditions separately, with the exception that the effect of group was only marginal in the CONF6 condition (F(2,275) = 2,56, p = .08), but showed the same pattern (i.e., less approach in HCs than in DEP/ANX and SUDs; p = .04 and .05, respectively). Within AV trials, we observed an effect of group (F(2,273) = 8.21, p < .001), reflecting greater avoidance behavior in HCs than in both SUDs (p < .001) and DEP/ANX (marginal; p = .07), and greater avoidance in DEP/ANX than in SUDs (p = .003). We note that the difference between HCs and DEP/ANX was significant in the full sample (p = .02), suggesting this marginal result was due to low power in the matched sample. We also note a marginal effect of sex (F(1,169) = 3.53, p = .06; suggesting greater avoidance in females). No significant effects were observed in APP trials, although we note a marginal effect of time (F(1,223) = 3.07, p = .08), suggesting greater approach at 1-year follow-up. We note that there was a main effect of group in the full sample for APP trials, reflecting greater approach behavior in HCs than in SUDs (p = .001) and greater approach in DEP/ANX than in SUDs (p = .02). In other cases, similar results were found in the full sample (see supplementary results).

As in our previous study, we also report analyses of within-subject choice variability within the Appendix, and confirm its expected relationship with our decision uncertainty model parameter. Within-subject variability also showed expected group differences and effects of time; that is, greater variability in SUDs than in HCs and DEP/ANX (strongest in the AV and CONF6 conditions), and reduced choice variability over time.

**Standard descriptive analyses in the full sample**

In identical LMEs to those preformed above, when averaging RTs across trial types there was a main effect of group (F(2,477) = 4.41, *p* = .01) and time (F(1,352) = 8.43, *p* = .004; significantly faster in the 1-year follow-up compared to baseline). Post-hoc contrasts revealed that RTs were significantly faster in HCs than in both SUDs (*p* = .003) and DEP/ANX (*p* = .04). Effects of sex and group (as well as their interaction with time) were non-significant. When analyzing separately by condition, the three conflict conditions only showed the effect of time (*p*s between .002 and .03). However, the two non-conflict conditions showed a different pattern. The AV condition showed a main effect of group (F(2,478) = 9.45, *p* < .001) and sex (F(1,457) = 5.48, *p* = .02; faster RTs in females), and only a marginal effect of time (F(1,357) = 3.60, *p* = .06), but no interactions between group and sex or time. The main effect of group reflected faster in RTs in HCs than in SUDs (*p* < .001) and DEP/ANX (*p* < .001). The APP condition only showed a significant effect of group (F(2,477) = 5.40, *p* = .005), reflecting faster RTs in HCs than both SUDs (*p* = .004) and DEP/ANX (marginally, *p* = .05). and marginally faster RTs in DEP/ANX than SUDs (*p* = .054).

**Table S1. Summary statistics for task reaction times (Mean (SD)) at baseline and follow-up**

| **Full Sample** | **HCs**  **(N = 49)** | **DEP/ANX**  **(N = 192)** | **SUDs**  **(N = 84)** |
| --- | --- | --- | --- |
| **Avoid Condition (Baseline)** | 1.19 (0.24) | 1.41 (0.34) | 1.43 (0.34) |
| **Avoid Condition (1-year follow-up)** | 1.20 (0.33) | 1.33 (0.37) | 1.40 (0.33) |
| **Approach Condition (Baseline)** | 1.11 (0.22) | 1.28 (0.35) | 1.23 (0.38) |
| **Approach Condition (1-year follow-up)** | 1.11 (0.27) | 1.20 (0.36) | 1.25 (0.38) |
| **Conflict: 2 points (Baseline)** | 1.20 (0.34) | 1.28 (0.36) | 1.26 (0.39) |
| **Conflict: 2 points (1-year follow-up)** | 1.14 (0.29) | 1.22 (0.39) | 1.26 (0.36) |
| **Conflict: 4 points (Baseline)** | 1.18 (0.34) | 1.26 (0.36) | 1.20 (0.35) |
| **Conflict: 4 points (1-year follow-up)** | 1.09 (0.28) | 1.17 (0.34) | 1.23 (0.41) |
| **Conflict: 6 points (Baseline)** | 1.20 (0.37) | 1.28 (0.36) | 1.20 (0.34) |
| **Conflict: 6 points (1-year follow-up)** | 1.09 (0.23) | 1.19 (0.35) | 1.22 (0.37) |
| **Propensity Matched** | **HCs**  **(N = 48)** | **DEP/ANX**  **(N = 121)** | **SUDs**  **(N = 29)** |
| **Avoid Condition (Baseline)** | 1.19 (0.24) | 1.37 (0.32) | 1.41 (0.33) |
| **Avoid Condition (1-year follow-up)** | 1.21 (0.33) | 1.29 (0.34) | 1.38 (0.32) |
| **Approach Condition (Baseline)** | 1.11 (0.22) | 1.25 (0.33) | 1.19 (0.36) |
| **Approach Condition (1-year follow-up)** | 1.11 (0.27) | 1.17 (0.34) | 1.19 (0.32) |
| **Conflict: 2 points (Baseline)** | 1.20 (0.34) | 1.26 (0.36) | 1.21 (0.33) |
| **Conflict: 2 points (1-year follow-up)** | 1.14 (0.29) | 1.18 (0.36) | 1.19 (0.30) |
| **Conflict: 4 points (Baseline)** | 1.18 (0.34) | 1.23 (0.33) | 1.15 (0.28) |
| **Conflict: 4 points (1-year follow-up)** | 1.09 (0.28) | 1.13 (0.32) | 1.16 (0.31) |
| **Conflict: 6 points (Baseline)** | 1.20 (0.37) | 1.26 (0.35) | 1.12 (0.24) |
| **Conflict: 6 points (1-year follow-up)** | 1.09 (0.23) | 1.16 (0.34) | 1.15 (0.36) |

In identical LMEs to those preformed above, average chosen runway position across the three conflict conditions showed greater approach behavior in males than females (*F*(1,450) = 9.71, *p* = .002), as well as an effect of group (*F*(2,472) = 7.67, *p* < .001) and a group by sex interaction (*F*(2,458) = 4.84, *p* = .008). The main effect reflected less approach behavior in HCs than in DEP/ANX and SUDs (*p* = .01 and *p* < .001, respectively), and less approach behavior in DEP/ANX than SUDs (*p* = .04). The interaction reflected the presence of all group differences stated above in females (*p*s < .001 to .004), but the absence of these effects in males (*p*s between .56 and .77). The same pattern was found when analyzing each conflict condition separately, with the exception that the difference between SUDs and DEP/ANX in CONF4 was only marginal (*p* = .06). Within AV trials, we observed an effect of group (*F*(2,476) = 17.00, *p* < .001), reflecting greater avoidance behavior in HCs than in both SUDs (*p* < .001) and DEP/ANX (*p* = .02). and greater avoidance in DEP/ANX than in SUDs (*p* < .001). Within APP trials, we observed an effect of group (*F*(2,472) = 6.00, *p* = .002), reflecting greater approach behavior in HCs than in SUDs (*p* = .001) and greater approach in DEP/ANX than in SUDs (*p* = .02).


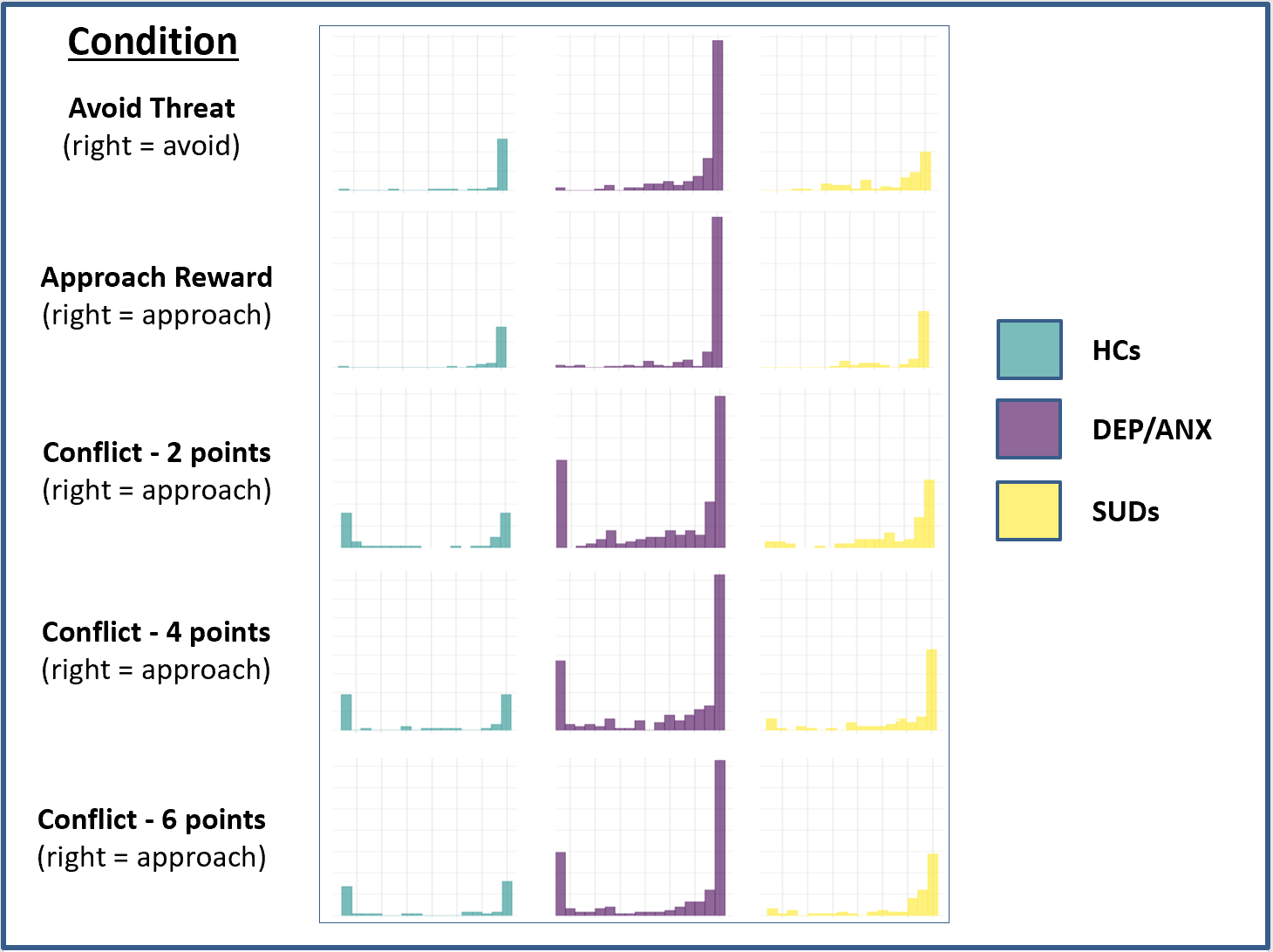
 **Figure S2.** Histograms showing mean runway position by group and condition. In the “Avoid Threat” condition, higher values (toward the right) on the x-axis indicate greater avoidance behavior. In all other conditions, higher values on the x-axis indicate greater approach behavior. As can be seen here, healthy controls (HCs) tend to approach vs. avoid completely (always move all the way toward the left or the right) and tend to do so in roughly equivalent proportions within the conflict conditions. In contrast, individuals with depression/anxiety (DEP/ANX) and substance use disorders (SUDs) tend to avoid less, and they more often choose values in the middle of the runway.

**Table S2. Summary statistics for chosen runway position (Mean (SD)) at baseline and follow-up^†^**

| **Full Sample** | **HCs**  **(N = 49)** | **DEP/ANX**  **(N = 192)** | **SUDs**  **(N = 84)** |
| --- | --- | --- | --- |
| **Avoid Condition (Baseline)** | 9.60 (0.81) | 9.06 (1.61) | 8.01 (2.24) |
| **Avoid Condition (1-year follow-up)** | 9.48 (1.50) | 9.19 (1.49) | 8.50 (1.78) |
| **Approach Condition (Baseline)** | 9.83 (0.42) | 9.25 (1.49) | 9.23 (1.19) |
| **Approach Condition (1-year follow-up)** | 9.65 (1.22) | 9.37 (1.54) | 9.21 (1.36) |
| **Conflict: 2 points (Baseline)** | 6.90 (3.16) | 7.12 (2.88) | 7.99 (2.36) |
| **Conflict: 2 points (1-year follow-up)** | 6.09 (3.68) | 7.21 (3.19) | 8.27 (2.31) |
| **Conflict: 4 points (Baseline)** | 7.19 (3.28) | 7.51 (2.86) | 8.22 (2.35) |
| **Conflict: 4 points (1-year follow-up)** | 6.25 (3.72) | 7.41 (3.21) | 8.38 (2.49) |
| **Conflict: 6 points (Baseline)** | 7.43 (3.25) | 7.67 (2.84) | 8.38 (2.32) |
| **Conflict: 6 points (1-year follow-up)** | 6.43 (3.71) | 7.51 (3.27) | 8.50 (2.40) |
| **Propensity Matched** | **HCs**  **(N = 48)** | **DEP/ANX**  **(N = 121)** | **SUDs**  **(N = 29)** |
| **Avoid Condition (Baseline)** | 9.60 (0.81) | 9.17 (1.54) | 8.19 (2.18) |
| **Avoid Condition (1-year follow-up)** | 9.47 (1.51) | 9.20 (1.51) | 8.76 (1.67) |
| **Approach Condition (Baseline)** | 9.83 (0.42) | 9.21 (1.60) | 9.30 (1.19) |
| **Approach Condition (1-year follow-up)** | 9.64 (1.23) | 9.43 (1.45) | 9.58 (0.98) |
| **Conflict: 2 points (Baseline)** | 6.90 (3.16) | 7.08 (2.95) | 7.94 (2.62) |
| **Conflict: 2 points (1-year follow-up)** | 6.18 (3.67) | 7.35 (3.20) | 8.17 (2.85) |
| **Conflict: 4 points (Baseline)** | 7.19 (3.28) | 7.49 (2.91) | 8.04 (2.69) |
| **Conflict: 4 points (1-year follow-up)** | 6.34 (3.71) | 7.51 (3.24) | 8.11 (3.04) |
| **Conflict: 6 points (Baseline)** | 7.43 (3.25) | 7.62 (2.90) | 8.36 (2.66) |
| **Conflict: 6 points (1-year follow-up)** | 6.51 (3.71) | 7.53 (3.30) | 8.06 (2.91) |

**^†^ Higher values indicate greater approach behavior (i.e., toward the points). In the avoid condition, higher values indicate runway positions closer to the positive stimulus.**

**Post-hoc analyses of within-subject variability**

Based on the group differences we observed in decision uncertainty, as in our previous study we performed post-hoc analyses of descriptive measures of behavioral variability. While not examined in previous studies, longitudinally stable differences in behavioral variability are implied by our modeling results. **Table S3** displays the within-subject SDs across trials by task condition and group comparisons. As can be seen there, within-subject choice variability in both the full and propensity-matched samples differed significantly between groups in a manner following a similar pattern seen in the decision uncertainty parameter.

*Matched Sample*

In LMEs examining variability (standard deviation) in chosen runway position across the three conflict conditions, there was a main effect of time (*F*(1,242) = 12.00, *p* < .001; less variability at 1-year follow-up). There was no main effect group or sex, or interactions between group and either sex or time). The same pattern was found when analyzing CONF2 and CONF4 separately; however, in CONF6 there was an additional effect of group (*F*(2,267) = 5.16, *p* = .006), reflecting greater variability in SUDs than in HCs and DEP/ANX (*p* = .003 and .005, respectively). Within AV trials, we observed an effect of group (*F*(2,272) = 13.48, *p* < .001), reflecting greater variability in SUDs than in both DEP/ANX and HCs (*p* < .001 each) and greater variability in DEP/ANX than in HCs (*p* = .01). There were no other main effects or interactions, although we note a marginal effect of sex (*F*(1,268) = 3.67, *p* = .06) suggesting greater variability in males. Within APP trials, we observed a main effect of time (*F*(1,236) = 6.61, *p* = .01; less variability at 1-year follow-up). We also note a marginal effect of group (*F*(2,272) = 2.44, *p* = .09), suggesting the effect of group reflected greater variability in SUDs than in both HCs (*p* = .03). This effect was significant in the full sample, suggesting this marginal result may be due to low power given the small sample size for SUDs in the matched sample.

*Full Sample*

In LMEs examining variability (standard deviation) in chosen runway position across the three conflict conditions, there were main effects of group (*F*(2,462) = 6.30, *p* = .002) and time (*F*(1,392) = 15.85, *p* < .001; less variability at 1-year follow-up). The main effect of group reflected greater variability in SUDs than in HCs and DEP/ANX (*p* = .001 and *.006*, respectively). There was no main effect of sex or interactions between group and either sex or time. The same pattern was found when analyzing each conflict condition separately, with the exception that the difference between SUDs and DEP/ANX was only marginal in the CONF4 condition (*p* = .07). Within AV trials, we observed an effect of group (*F*(2,471) = 26.60, *p* < .001), reflecting greater variability in SUDs than in both DEP/ANX and HCs (*p* < .001 each) and greater variability in DEP/ANX than in HCs (*p* = .003). There were no other main effects or interactions. Within APP trials, we observed main effects of of group (*F*(2,468) = 8.81, *p* < .001) and time (*F*(1,384) = 4.60, *p* = .03; less variability at 1-year follow-up), The effect of group reflected greater variability in SUDs than in both HCs and DEP/ANX (*p* < .001 each).

The decision uncertainty parameter was also significantly correlated with within-subject SDs across conditions, as would be expected (see **Figure S3**).


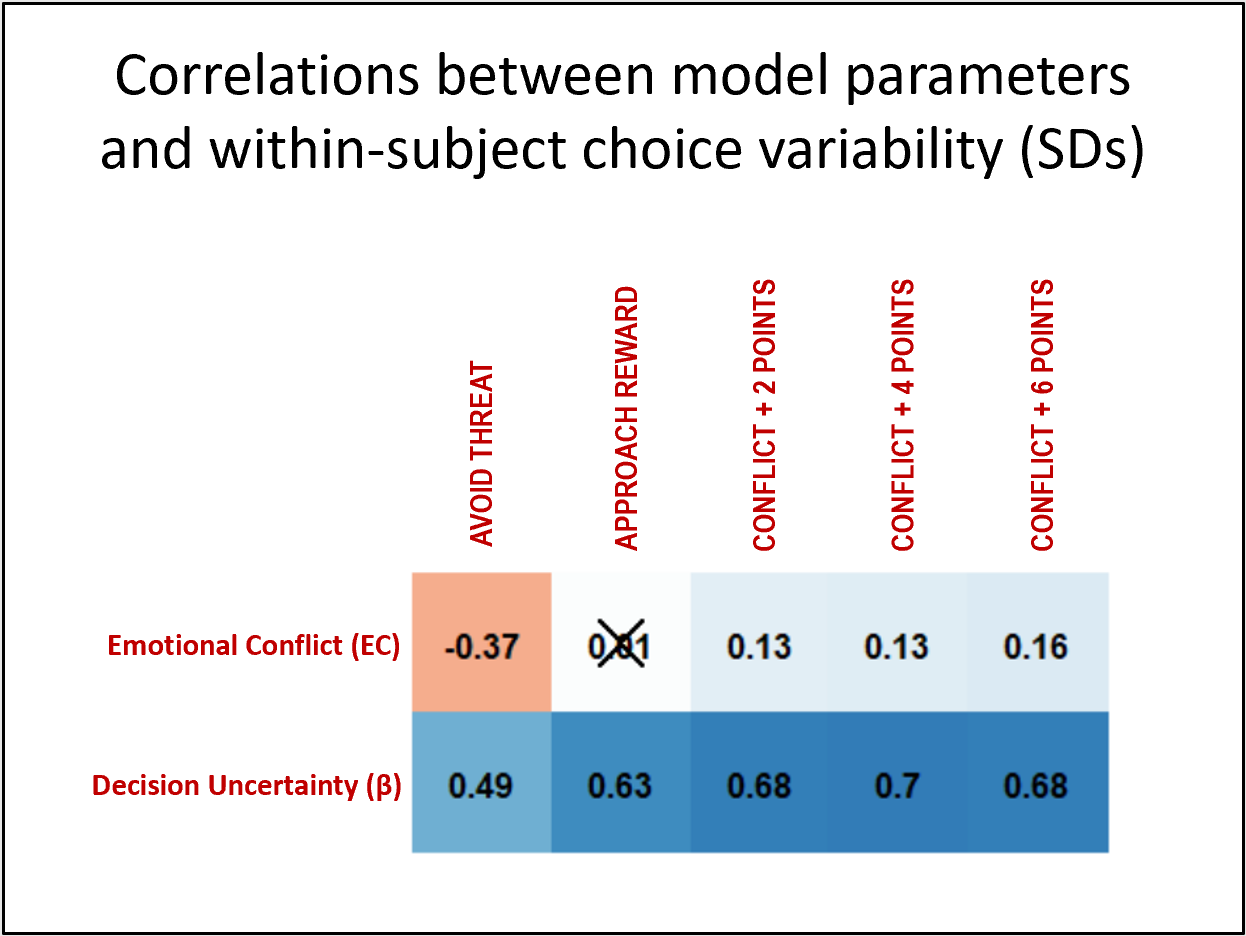


**Figure S3**. Pearson correlations between parameter estimates (full sample) and within-subject choice variability (per task condition). The ‘X’ over a correlation indicates a p-value greater than .05. Higher decision uncertainty is associated with greater variability, as expected. Emotional conflict is negatively associated with variability in the ‘avoid threat’, suggesting that those more driven to avoid threat are less uncertain about what to do.

**Table S3. Summary statistics for within-subject variability (SD) in chosen runway position (Mean (SD))**

| **Full Sample** | **HCs**  **(N = 49)** | **DEP/ANX**  **(N = 192)** | **SUDs**  **(N = 84)** |
| --- | --- | --- | --- |
| **Avoid Condition (Baseline)** | 0.64 (0.99) | 0.89 (1.17) | 1.48 (1.27) |
| **Avoid Condition (1-year follow-up)** | 0.40 (0.91) | 0.87 (1.15) | 1.54 (1.26) |
| **Approach Condition (Baseline)** | 0.38 (0.75) | 0.73 (1.11) | 0.96 (1.15) |
| **Approach Condition (1-year follow-up)** | 0.41 (0.92) | 0.56 (1.05) | 0.80 (1.21) |
| **Conflict: 2 points (Baseline)** | 1.15 (1.27) | 1.38 (1.25) | 1.38 (1.24) |
| **Conflict: 2 points (1-year follow-up)** | 0.74 (1.14) | 0.94 (1.23) | 1.16 (1.21) |
| **Conflict: 4 points (Baseline)** | 0.80 (1.01) | 1.09 (1.19) | 1.13 (1.17) |
| **Conflict: 4 points (1-year follow-up)** | 0.53 (1.08) | 0.84 (1.18) | 0.85 (1.16) |
| **Conflict: 6 points (Baseline)** | 0.86 (1.09) | 1.11 (1.26) | 1.20 (1.22) |
| **Conflict: 6 points (1-year follow-up)** | 0.60 (1.08) | 0.73 (1.12) | 1.07 (1.25) |
| **Propensity Matched** | **HCs**  **(N = 48)** | **DEP/ANX**  **(N = 121)** | **SUDs**  **(N = 29)** |
| **Avoid Condition (Baseline)** | 0.64 (0.99) | 0.84 (1.17) | 1.48 (1.18) |
| **Avoid Condition (1-year follow-up)** | 0.41 (0.92) | 0.85 (1.17) | 1.45 (1.24) |
| **Approach Condition (Baseline)** | 0.38 (0.75) | 0.71 (1.09) | 0.77 (1.09) |
| **Approach Condition (1-year follow-up)** | 0.42 (0.92) | 0.55 (1.07) | 0.47 (0.85) |
| **Conflict: 2 points (Baseline)** | 1.15 (1.27) | 1.32 (1.25) | 1.22 (1.29) |
| **Conflict: 2 points (1-year follow-up)** | 0.75 (1.15) | 0.90 (1.17) | 0.94 (1.08) |
| **Conflict: 4 points (Baseline)** | 0.80 (1.01) | 1.06 (1.14) | 0.97 (1.26) |
| **Conflict: 4 points (1-year follow-up)** | 0.54 (1.09) | 0.78 (1.15) | 0.57 (1.04) |
| **Conflict: 6 points (Baseline)** | 0.86 (1.09) | 1.04 (1.22) | 0.93 (1.26) |
| **Conflict: 6 points (1-year follow-up)** | 0.57 (1.07) | 0.68 (1.09) | 1.04 (1.26) |

**Group differences in post-task self-report questions**

As additional confirmation of behavioral results, we also used LMEs to assess group differences in self-reported approach-avoidance motivations, anxiety, and decision difficulty during the task. Descriptive values for all post-task self-report questions by group are shown in the **Table S4**.

*Matched Sample*

For self-reported task anxiety, no significant effects were observed. Although, we note marginal effects of sex, group, and time (*p*s between .07 and .09), which were significant in the full sample (greater anxiety in females, greater anxiety at baseline, and greater anxiety in DEP/ANX than SUDs; see below).

For self-reported decision difficulty on the task, there were main effects of time (F(1,240) = 16.64, p < .001; greater at baseline) and group F(2,271) = 3.8, *p* = .02). The main effect of group reflected lower decision difficulty in HCs than in both SUDs and DEP/ANX (*p* = .03 and .01, respectively). Similar results were found in the full sample (see below).

For self-reported approach motivations on the task, there was a main effects of sex (F(1,268) = 5.36, p = .02; less approach in females). We also note a marginal effect of group (*p* = .06), which was significant in the full sample (see below). This suggested less approach motivations in HCs than in both SUDs and DEP/ANX.

For self-reported avoidance motivations on the task, no significant effects were observed. However, we note marginal effects of sex and its interaction with group (*p*s = .05 and .06, respectively), which were significant in the full sample (indicating greater avoidance in females; and greater avoidance in HCs than SUDs and DEP/ANX in females, while no groups differed in males; see below).

*Full Sample*

For self-reported anxiety, there were main effects of sex (*F*(1,448) = 7.16, *p* = .007; greater in females), time (*F*(1,380) = 7.05, *p* = .008; greater at baseline), and group *F*(2,470) = 3.52, *p* = .03). The main effect of group reflected greater anxiety in DEP/ANX than SUDs (*p* = .008).

For self-reported decision difficulty, there were main effects of time (*F*(1,382) = 20.60, *p* < .001; greater at baseline) and group *F*(2,468) = 5.0, *p* = .007). The main effect of group reflected lower decision difficulty in HCs than in both SUDs and DEP/ANX (*p* = .002 and .02, respectively).

For self-reported approach behavior, there were main effects of sex (*F*(1,448) = 7.18, *p* = .008; less approach in females) and group (*F*(2,470) = 5.55, *p* = .004). The main effect of group reflected less approach behavior in HCs than in both SUDs and DEP/ANX (*p* = .001 and *.04*, respectively).

For self-reported avoidance behavior, there were main effects of sex (*F*(1,447) = 8.04, *p* = .005; greater in females), group (*F*(2,468) = 3.12, *p* = .04), and their interaction (*F*(2,455) = 3.33, *p* = .04). The main effect of group reflected greater avoidance in HCs than SUDs (*p* = .02). The interaction reflected that HCs showed greater avoidance than SUDs and DEP/ANX in females (*p < .001* and *p* = .005, respectively), while no groups differed in males.

**Table S4. Task-specific self-report questionnaire summary statistics (Mean (SD)) at baseline and follow-up***

| **Full Sample** | **HCs**  **(N = 49)** | **DEP/ANX**  **(N = 192)** | **SUDs**  **(N = 84)** |
| --- | --- | --- | --- |
| **1. I found the POSITIVE pictures enjoyable (Baseline)** | 5.08 (1.87) | 5.07 (1.68) | 5.25 (1.58) |
| **1. I found the POSITIVE pictures enjoyable (1-year follow-up)** | 5.27 (1.51) | 4.99 (1.55) | 5.01 (1.56) |
| **2. The NEGATIVE pictures made me feel anxious Or uncomfortable (Baseline)** | 4.56 (1.83) | 4.81 (1.97) | 4.14 (2.05) |
| **2. The NEGATIVE pictures made me feel anxious Or uncomfortable (1-year follow-up)** | 4.00 (1.66) | 4.52 (2.05) | 3.79 (1.84) |
| **3. I often found it difficult to decide which outcome I wanted (Baseline)** | 2.17 (1.65) | 2.49 (1.79) | 2.58 (1.69) |
| **3. I often found it difficult to decide which outcome I wanted (1-year follow-up)** | 1.47 (0.92) | 2.08 (1.65) | 2.12 (1.65) |
| **4. I always tried to move ALL THE WAY TOWARDS the outcome with the LARGEST REWARD POINTS (Baseline)** | 4.42 (2.60) | 4.48 (2.44) | 5.15 (2.04) |
| **4. I always tried to move ALL THE WAY TOWARDS the outcome with the LARGEST REWARD POINTS (1-year follow-up)** | 3.96 (2.73) | 4.78 (2.55) | 5.46 (1.97) |
| **5. I always tried to move ALL THE WAY AWAY FROM the outcome with the NEGATIVE PICTURE/SOUNDS (Baseline)** | 3.27 (2.52) | 3.05 (2.23) | 2.58 (2.06) |
| **5. I always tried to move ALL THE WAY AWAY FROM the outcome with the NEGATIVE PICTURE/SOUNDS (1-year follow-up)** | 3.76 (2.73) | 3.30 (2.41) | 2.54 (2.00) |
| **6. When a NEGATIVE picture and sound were displayed, I kept my eyes open and looked at the picture (Baseline)** | 5.33 (1.96) | 5.15 (1.99) | 5.96 (1.56) |
| **6. When a NEGATIVE picture and sound were displayed, I kept my eyes open and looked at the picture (1-year follow-up)** | 5.12 (2.26) | 4.95 (2.14) | 5.71 (1.44) |
| **7. When a NEGATIVE picture and sound were displayed, I tried to think about something unrelated to the picture to distract myself (Baseline)** | 3.52 (2.01) | 3.20 (2.04) | 2.49 (1.73) |
| **7. When a NEGATIVE picture and sound were displayed, I tried to think about something unrelated to the picture to distract myself (1-year follow-up)** | 3.27 (2.03) | 3.46 (2.13) | 3.11 (1.99) |
| **8. When a NEGATIVE picture and sound were displayed, I tried other strategies to manage emotions triggered by the pictures (Baseline)** | 3.50 (1.79) | 3.58 (2.08) | 2.87 (1.81) |
| **8. When a NEGATIVE picture and sound were displayed, I tried other strategies to manage emotions triggered by the pictures (1-year follow-up)** | 3.43 (2.01) | 3.72 (2.12) | 3.46 (1.99) |
| **Propensity Matched** | **HCs**  **(N = 48)** | **DEP/ANX**  **(N = 121)** | **SUDs**  **(N = 29)** |
| **1. I found the POSITIVE pictures enjoyable (Baseline)** | 5.08 (1.87) | 5.07 (1.72) | 5.07 (1.69) |
| **1. I found the POSITIVE pictures enjoyable (1-year follow-up)** | 5.31 (1.49) | 4.92 (1.58) | 4.97 (1.72) |
| **2. The NEGATIVE pictures made me feel anxious Or uncomfortable (Baseline)** | 4.56 (1.83) | 4.73 (1.91) | 3.55 (2.16) |
| **2. The NEGATIVE pictures made me feel anxious Or uncomfortable (1-year follow-up)** | 3.96 (1.65) | 4.43 (2.07) | 3.66 (1.84) |
| **3. I often found it difficult to decide which outcome I wanted (Baseline)** | 2.17 (1.65) | 2.41 (1.78) | 2.28 (1.62) |
| **3. I often found it difficult to decide which outcome I wanted (1-year follow-up)** | 1.48 (0.92) | 1.97 (1.52) | 1.86 (1.66) |
| **4. I always tried to move ALL THE WAY TOWARDS the outcome with the LARGEST REWARD POINTS (Baseline)** | 4.42 (2.60) | 4.55 (2.40) | 4.97 (2.21) |
| **4. I always tried to move ALL THE WAY TOWARDS the outcome with the LARGEST REWARD POINTS (1-year follow-up)** | 4.02 (2.72) | 4.84 (2.56) | 5.41 (2.29) |
| **5. I always tried to move ALL THE WAY AWAY FROM the outcome with the NEGATIVE PICTURE/SOUNDS (Baseline)** | 3.27 (2.52) | 2.83 (2.13) | 2.86 (2.18) |
| **5. I always tried to move ALL THE WAY AWAY FROM the outcome with the NEGATIVE PICTURE/SOUNDS (1-year follow-up)** | 3.81 (2.73) | 3.17 (2.44) | 2.41 (2.15) |
| **6. When a NEGATIVE picture and sound were displayed, I kept my eyes open and looked at the picture (Baseline)** | 5.33 (1.96) | 5.14 (2.01) | 5.83 (1.67) |
| **6. When a NEGATIVE picture and sound were displayed, I kept my eyes open and looked at the picture (1-year follow-up)** | 5.21 (2.20) | 5.00 (2.13) | 5.59 (1.57) |
| **7. When a NEGATIVE picture and sound were displayed, I tried to think about something unrelated to the picture to distract myself (Baseline)** | 3.52 (2.01) | 3.14 (2.03) | 2.31 (1.71) |
| **7. When a NEGATIVE picture and sound were displayed, I tried to think about something unrelated to the picture to distract myself (1-year follow-up)** | 3.23 (2.03) | 3.50 (2.14) | 2.93 (2.15) |
| **8. When a NEGATIVE picture and sound were displayed, I tried other strategies to manage emotions triggered by the pictures (Baseline)** | 3.50 (1.79) | 3.61 (2.12) | 2.62 (1.82) |
| **8. When a NEGATIVE picture and sound were displayed, I tried other strategies to manage emotions triggered by the pictures (1-year follow-up)** | 3.48 (2.00) | 3.82 (2.11) | 3.66 (2.14) |

***All ratings were on a scale of 1-7.**

**Exploratory analysis of longitudinal symptom prediction**

Our result in the main text that higher EC values at baseline predicted larger increases (or smaller decreases) in behavioral inhibition over time prompted us to further assess effects of group and session on BIS scores (Baseline: HCs, mean = 18.1, SD = 3.2; DEP/ANX, mean = 23.7, SD = 3.4;. SUDs, mean = 21.0, SD = 3.9; Follow-up: HCs, mean = 18.1, SD = 3.5; DEP/ANX, mean = 23.1, SD = 3.7;. SUDs, mean = 19.4, SD = 4.2). An LME revealed main effects of group (F(2,483) = 75.38, p < .001) and time (F(1,358) = 10.09, p < .001; greater scores at baseline), and a group by time interaction (F(2,361) = 4.51, p = .01). The group effect reflected greater BIS scores in DEP/ANX than SUDS and HCs, and greater BIS scores in SUDS than HCs (p < .001 each). The interaction reflected significant decreases in BIS scores in DEP/ANX and SUDs (p = .02 and p < .001, respectively), but no change in HCs.

As the Tulsa 1000 has a large number of clinically relevant measures, for hypothesis generation purposes we also conducted an exploratory analysis of partial correlations between baseline task measures and several available measures (after accounting for their baseline values), including Patient-Reported Outcomes Measurement Information System (PROMIS) depression and anxiety scales (22), the Behavioral Activation/Inhibition (BIS/BAS) scales (23), the PANAS positive and negative affect scales (24), the Anxiety Sensitivity Index (ASI; (25)), the temporal experience of (anticipatory and consummatory) pleasure scales (TEPS; (26)), and the state-trait anxiety inventory (STAI) scales (27). As can be seen in **Figure S4**, task measures did not show strong predictive relationships with changes in clinical measures over time. However, both lower EC parameter values at baseline and less behavioral avoidance (chosen runway position) in the conflict conditions at baseline weakly predicted reductions in behavioral inhibition over time. Stronger avoidance in the avoid (AV) condition at baseline also weakly predicted greater reductions in negative affect (PANAS) over time (but, as noted in the main text, this was not significant within our LMEs accounting for age, sex, and WRAT scores). Although not shown in the figure, no predictive relationships were found between any task measure and changes in the PHQ, OASIS, or DAST screening measures (i.e., for depression, anxiety, and substance use severity, respectively).


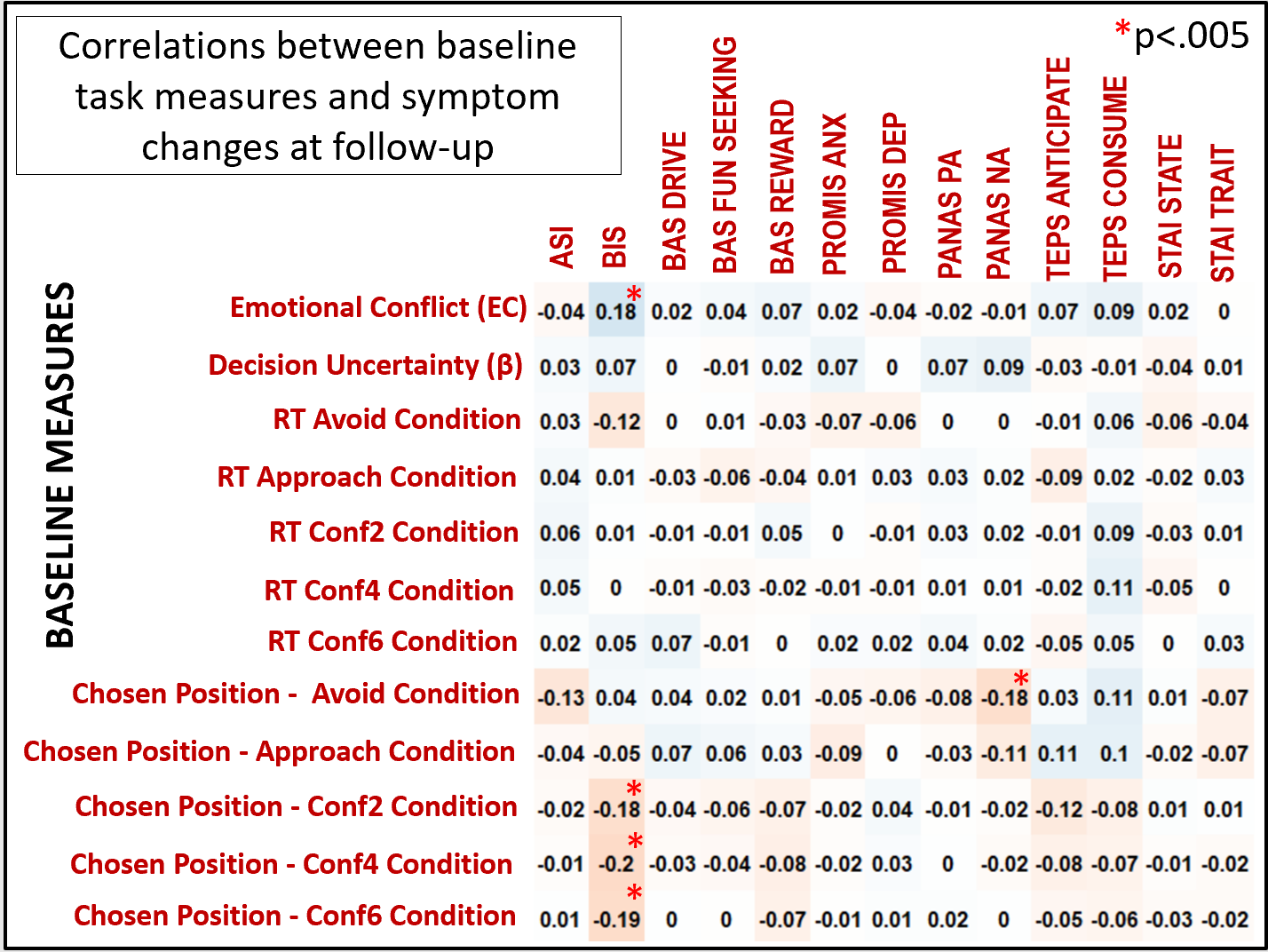


**Figure S4.** Exploratory analysis of the ability of baseline task measures (left) to predict longitudinal changes in symptoms from baseline to follow-up (top; after accounting for differences in baseline values). Red stars indicate uncorrected p-values < .005. Measures include the anxety sensitivity index (ASI), behavioral inhibition and activation scales (BIS/BAS), PROMIS depression and anxiety scales, PANAS positive and negative affect (PA/NA) scales, the temporal experience of (anticipatory and consummatory) pleasure scales (TEPS), and the state-trait anxiety inventory (STAI) scales.

**Additional information about short-term test-retest sample**

Descriptive behavioral measures for the additional healthy control sample of 30 participants are shown in **Table S5**. This sample had a shorter test-retest interval of approximately 2-3 weeks (mean = 17.10 days; SD = 5.18; range: 11-32). There were a few differences between the task used in this sample and the version described in the main text (for more details, see (28)). First, it was conducted during functional magnetic resonance imaging. Second, participants used a joystick instead of key press to move the avatar. Third, the task had 90 trials (as opposed to 60 in the version described in the main text. As a result of having more trials, the avatar could also start in any position on the runway across trials in counterbalanced fashion. Finally, in place of 0, 2, 6, or 6 points per trial, participants were offered small monetary rewards (i.e., 0, 2, 4, or 6 United States’ cents).

**Table S5. Descriptive results for short-term test-retest sample**

| **Behavioral Measure** | **Time 1** | | **Time 2** | |
| --- | --- | --- | --- | --- |
| **Model Parameters** | **Mean** | **SD** | **Mean** | **SD** |
| **Emotion Conflict (EC)** | 3.59 | 2.93 | 3.64 | 2.84 |
| **Decision Uncertainty (β)** | 2.33 | 2.42 | 2.08 | 2.01 |
| **Chosen Runway Position** | **Mean** | **SD** | **Mean** | **SD** |
| **Approach Condition** | 8.91 | 0.15 | 8.90 | 0.16 |
| **Avoid Condition** | 8.88 | 0.21 | 8.90 | 0.22 |
| **Conflict: 2 points** | 5.70 | 3.28 | 5.35 | 3.60 |
| **Conflict: 4 points** | 6.07 | 3.53 | 5.92 | 3.51 |
| **Conflict: 6 points** | 6.42 | 3.38 | 6.26 | 3.53 |
| **Reaction Time** | **Mean** | **SD** | **Mean** | **SD** |
| **Approach Condition** | 0.95 | 0.15 | 0.87 | 0.14 |
| **Avoid Condition** | 1.04 | 0.17 | 0.96 | 0.21 |
| **Conflict: 2 points** | 1.04 | 0.29 | 0.92 | 0.22 |
| **Conflict: 4 points** | 1.02 | 0.21 | 0.90 | 0.21 |
| **Conflict: 6 points** | 0.97 | 0.22 | 0.89 | 0.19 |
| **All trials** | 1.00 | 0.18 | 0.90 | 0.16 |

**References**

1. Technically, we did fix a related inverse temperature parameter (α) for action selection that is included in active inference models to a value of 16, which allowed for plausible levels of indeterminacy in selecting an action after inferring posterior policy values. [↑](#footnote-ref-2)
